# Differential genetic interactions of yeast stress response MAPK pathways

**DOI:** 10.15252/msb.20145606

**Published:** 2015-04-17

**Authors:** Humberto Martin, Michael Shales, Pablo Fernandez-Piñar, Ping Wei, Maria Molina, Dorothea Fiedler, Kevan M Shokat, Pedro Beltrao, Wendell Lim, Nevan J Krogan

**Affiliations:** 1 Departamento de Microbiología II, Facultad de Farmacia, Universidad Complutense de Madrid and Instituto Ramón y Cajal de Investigaciones Sanitarias (IRYCIS)Madrid, Spain; 2 Department of Cellular and Molecular Pharmacology, University of CaliforniaSan Francisco, CA, USA; 3 Center for Quantitative Biology and Peking-Tsinghua Center for Life Sciences, Peking UniversityBeijing, China; 4 Department of Chemistry, Princeton UniversityPrinceton, NJ, USA; 5 Chemistry and Chemical Biology Graduate Program, University of CaliforniaSan Francisco, CA, USA; 6 European Molecular Biology Laboratory, European Bioinformatics InstituteCambridge, UK; 7 iBiMED and Department of Health Sciences, University of AveiroAveiro, Portugal; 8 Howard Hughes Medical Institute, University of CaliforniaSan Francisco, CA, USA; 9 Center for Systems and Synthetic Biology, University of CaliforniaSan Francisco, CA, USA; 10 California Institute for Quantitative Biosciences, QB3San Francisco, CA, USA; 11 J. David Gladstone InstitutesSan Francisco, CA, USA

**Keywords:** cell wall integrity, genetic interactions, osmotic shock, stress response

## Abstract

Genetic interaction screens have been applied with great success in several organisms to study gene function and the genetic architecture of the cell. However, most studies have been performed under optimal growth conditions even though many functional interactions are known to occur under specific cellular conditions. In this study, we have performed a large-scale genetic interaction analysis in *Saccharomyces cerevisiae* involving approximately 49 × 1,200 double mutants in the presence of five different stress conditions, including osmotic, oxidative and cell wall-altering stresses. This resulted in the generation of a differential E-MAP (or dE-MAP) comprising over 250,000 measurements of conditional interactions. We found an extensive number of conditional genetic interactions that recapitulate known stress-specific functional associations. Furthermore, we have also uncovered previously unrecognized roles involving the phosphatase regulator Bud14, the histone methylation complex COMPASS and membrane trafficking complexes in modulating the cell wall integrity pathway. Finally, the osmotic stress differential genetic interactions showed enrichment for genes coding for proteins with conditional changes in phosphorylation but not for genes with conditional changes in gene expression. This suggests that conditional genetic interactions are a powerful tool to dissect the functional importance of the different response mechanisms of the cell.

## Introduction

Cells need to constantly evaluate and adapt to changes in environmental conditions. Variation in these conditions can result in suboptimal growth, and therefore, cells use complex response pathways that sense environmental changes and promote the appropriate response resulting in adapted cellular states. MAPK (mitogen-activated protein kinase) pathways are widely used through evolution to perform this essential function (Raman *et al*, 2007). Changes in environmental conditions are commonly detected by sensors located at the cell surface, and the signal is transduced by GTPase nodes to MAPK phosphorylation cascades. These cascades are characterized by a three-tiered module comprising a MAPK kinase kinase (MAPKKK), a MAPK kinase (MAPKK) and the MAPK itself, whose activation results from the sequential phosphorylation of each component kinase in turn (Marshall, 1994). Once activated, MAPKs target different downstream effectors ranging from cytoskeletal proteins to transcription factors that lead to changes in transcriptional programs (Yoon & Seger, 2006). The importance of phosphorylation in the transmission of the signal makes protein phosphatases the main negative regulators of signalling through MAPK pathways (Martín *et al*, 2005).

The budding yeast *S. cerevisiae* uses MAPK pathways to adapt to a wide variation in physical environmental conditions and chemical stimuli, such as osmotic stress or agents that disrupt the integrity of the cell wall. Whereas the high osmolarity glycerol (HOG) pathway is essential for an appropriate response and adaptation to hyperosmolarity, the cell wall integrity pathway (CWI) detects and responds to the cell wall stress that occurs under normal growth conditions or through environmental change (Levin, 2011; Saito & Posas, 2012). Due to the conservation of MAPK pathways, yeast studies have provided key advances in identifying components and elucidating molecular mechanisms underlying eukaryotic cellular signalling. Large-scale gene expression studies have been used to identify the transcriptional responses to different sets of conditions (Gasch & Werner-Washburne, 2002), and proteomic approaches have contributed to our understanding of MAPK-mediated phosphorylation (Soufi *et al*, 2009; Mascaraque *et al*, 2013). These regulatory networks result in functional inter-dependencies that can also be studied by large-scale genetic approaches. For example, chemical genetic studies have been used to identify genes that, when knocked out, increase sensitivity to external stress factors (Hillenmeyer *et al*, 2008). In addition, these regulatory networks have been analysed using quantitative genetic interaction mapping (Fiedler *et al*, 2009).

Genetic interactions quantify the extent by which the phenotype of a double mutant deviates from the expected combination of the individual mutations (Boone *et al*, 2007; Mani *et al*, 2008; Beltrao *et al*, 2010). In yeast, fitness measurement and knock-out libraries have been extensively used to measure genetic interactions. If a double mutant grows better than expected, then a positive or alleviating genetic interaction exists between the two genes. Conversely, it is said that a negative or aggravating genetic interaction exists when the double mutant grows worse than expected based on the fitness of the single mutants. It has been previously shown that quantitative genetic interaction mapping can be used to study the functional organization of regulatory networks in unstressed conditions (Tong *et al*, 2001; Pan *et al*, 2004; Lehner *et al*, 2006; Collins *et al*, 2007; Roguev *et al*, 2008; Typas *et al*, 2008; Costanzo *et al*, 2010; Braberg *et al*, 2013). However, it is known that these genetic inter-dependencies can change with variations in external conditions (Harrison *et al*, 2007; St Onge *et al*, 2007; Musso *et al*, 2008). We have previously developed a large-scale approach to map differential genetic interactions, termed dE-MAP, and have applied this method to study changes in genetic interactions in the presence of DNA-damaging agents (Bandyopadhyay *et al*, 2010; Ideker & Krogan, 2012). However, little is known about the specificity of differential genetic interactions towards distinct environmental perturbations. To study this, we applied the dE-MAP strategy to a diverse set of five stress conditions including osmotic, oxidative and cell wall stress agents. We observed a large number of conditional dependent genetic interactions that are specific and reflect previously known conditional dependent functional interactions. We have additionally identified many novel functional conditional gene–gene and gene–complex associations. Finally, we have compared osmotic differential genetic interactions with large-scale condition-dependent phosphoproteomics and gene expression information to dissect the contribution of these different types of regulation to the conditional fitness measurements.

## Results and Discussion

### Quantitative differential genetic interactions of stress response pathways

**Figure 1:**
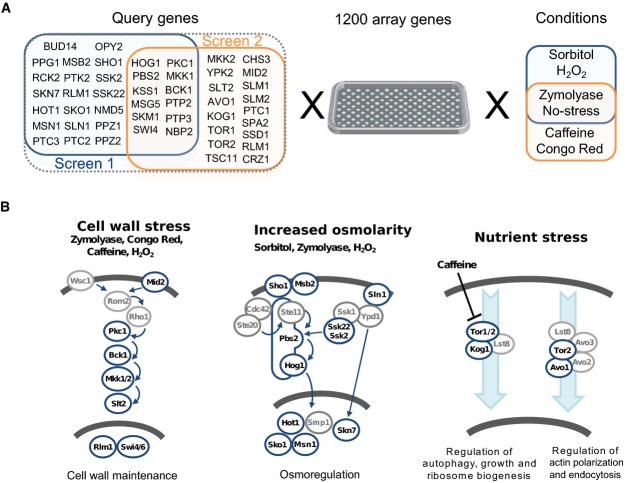
A differential epistatic interaction map (dE-MAP) of *Saccharomyces cerevisiae* MAPK stress pathways

A quantitative differential genetic map, or dE-MAP, of yeast MAPK stress response pathways was constructed based on two experimental screens (Materials and Methods). A total of 49 signalling-related query genes covering different pathways were crossed with an array containing approximately 1,200 genes that broadly cover different yeast cellular complexes and processes (Fig1A and B). The query genes are comprised of stress-sensing proteins, kinases, phosphatases, transcription factors and a few additional adaptor proteins (Fig1A). Many of the components overlap with the known members of the cell wall integrity (CWI) and the high osmolarity glycerol (HOG) pathways. The double mutants were arrayed on agar plates either in optimal growth conditions or in the presence of five different agents to provide distinct stresses: sorbitol (SO) as high osmotic stress; H_2_O_2_ (OX) as oxidative stress; zymolyase (ZY) and Congo red (CR), both cell wall-altering agents and thus CWI-activating agents; and caffeine (CA) which in addition to stimulating the CWI pathway (Martin *et al*, 2000) seems to inhibit the TOR pathway (Kuranda *et al*, 2006). Therefore, some mutants affected in TORC1 and TORC2 complexes, which have distinct physiological functions (Loewith *et al*, 2002), were also included in the query mutant collection. ZY is also known to activate the HOG pathway, which is required for sequential CWI pathway activation. In fact, activation of the CWI pathway is independent of the main receptors operating in this pathway, Wsc1 and Mid2, but requires the Sho1 branch of the HOG pathway (Bermejo *et al*, 2008). Double mutant colony sizes were quantified in each of the conditions, normalized and analysed to calculate a quantitative genetic interaction score (*S*-score) (Collins *et al*, 2006, 2010). A total of 343,200 genetic interaction scores were measured for the generation of this dE-MAP which allowed us to test for approximately 257,000 differential interactions. Biological replicates for *S*-scores derived from unstressed and for ZY-treated cells showed correlation coefficients similar to previous genetic interaction screens (Collins *et al*, 2007) (Supplementary Fig S1).

A total of 49 deletion query genes related to MAPK signalling (i.e. kinases, phosphatases, transcription factors, adaptors) were crossed against a deletion array of approximately 1,200 genes. The double mutants were screened in five different stress conditions.

The selected query genes broadly cover the cell wall integrity (CWI) and high osmolarity glycerol (HOG) pathways as well as a few members of the target of rapamycin (TOR) pathway. Genes in grey circles were not selected for screening.

**Figure 2:**
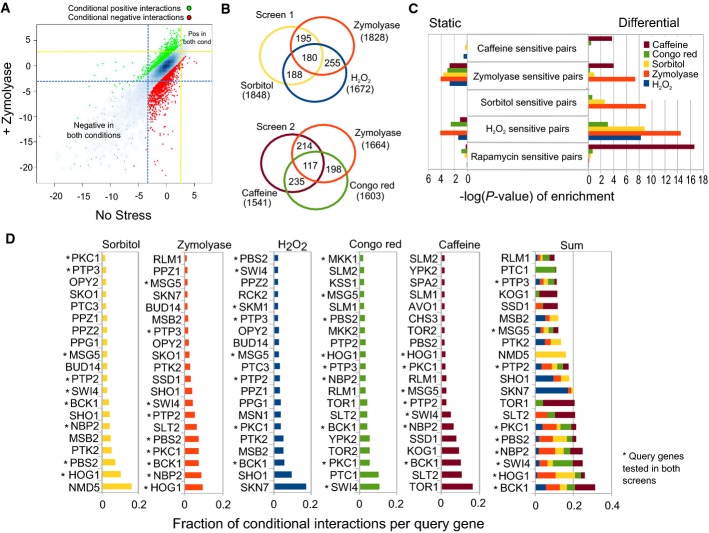
Differential genetic interactions are extensive and specific and recapitulate previously known functional associations

The significant genetic interactions (here defined as |*S*-score| ≥ 3) constitute the ‘static’ networks obtained from both stressed and unstressed conditions. In order to estimate the reproducibility of ‘static’ genetic interactions at this *S*-score cut-off, we compared biological replicates for two conditions (no-stress and ZY) for the double mutants that were screened in both conditions. For the normal growth condition, 53 and 55% of genetic interactions identified in screen 1 and screen 2, respectively, are detected at the same or higher threshold in the replicate. For the ZY condition, 51 and 63% of genetic interactions observed in screen 1 and screen 2, respectively, are detected at the same or higher threshold in the replicate. We have also compared the genetic interactions reported here with a previous study that contained many of the signalling same gene pairs (Fiedler *et al*, 2009). For the normal growth condition, 33 and 42% of genetic interactions identified in screen 1 and screen 2, respectively, were also found in that previous study. These results are in line with previous comparisons of genetic interaction screens (Ryan *et al*, 2012). We then defined as the conditional or differential genetic interactions those that significantly change from the unstressed to the stress conditions using an approach we have previously described (Bandyopadhyay *et al*, 2010). The variance of *S*-scores was determined as a function of its magnitude for the unstressed condition, and a *z*-score value was calculated for each gene pair in the presence of each stress condition (Materials and Methods). An absolute value *z*-score cut-off of 2 was used to define the significant differential genetic interactions. We show in Fig2A a scatterplot with *S*-scores for the same gene pairs screened in the presence or absence of ZY. The gene pairs with a conditional genetic interaction *z*-score higher than 2 are highlighted in the Figure (Fig2A, red and green dots).

Scatterplot of the genetic interaction *S*-scores in the presence and absence of zymolyase. Strong genetic interactions were defined as |*S*-score| ≥ 3, and strong differential interactions were defined as |*z*-score| ≥ 2. Dashed lines delineate the *S*-score threshold values for strong positive (yellow line) and strong negative (blue line) static interactions. The differential interactions were highlighted in green (differential positive) and red (differential negative).

Venn diagrams with the total number and overlaps of differential genetic interactions in each condition.

Gene deletions causing sensitivity to different stress conditions were collected from previous chemical genetic studies. The enrichment of pairs of these sensitivity genes was calculated for each static and differential genetic interaction network. The significance of the enrichment tests was converted to –log(*P*-value).

For each condition, we calculated the fraction of the total differential interactions explained by each of the query strains. The query strains were ranked according to this metric, and the top 20 most interacting strains are represented here.

### Benchmarking of the conditional genetic interaction networks

In order to assess the quality of the conditional genetic interactions determined in this study, we have assessed reproducibility across biological replicates and validated a subset of interactions using viability spot assays. For the ZY condition, genetic interactions that exist in both biological replicates display a correlation of 0.46 (Supplementary Fig S2). We have also tested the reproducibility of the ZY conditional interactions at the threshold selected. Using this condition, 44 and 36% of conditional genetic interactions identified in screen 1 and screen 2, respectively, are detected at the same or higher threshold in the replicate. In order to further validate the conditional genetic interactions experimentally, we re-generated a set of 31 double mutants, including 21 pairs with *HOG1* and 10 with *SLT2,* and compared the viability of the single and double mutants in stress and no stress using spot assays (total of 93 conditional interactions) (Supplementary Fig S3). For all but 2 of the 31 pairs, the double mutant did not appear to have a genetic interaction in absence of stress, consistent with the no-stress *S*-score value for 90% of the pairs. We scored the change in genetic interaction in a qualitative schema with five groups from strongly conditional negative to strongly conditional positive (– –/−/n/+/++). Two gene pairs displayed negative interactions in the absence of stress in the spot assays (*HOG1*-*PHO80* and *HOG1*-*VPS9*). For these, we could not easily score the change in genetic interaction in SO or ZY in the spot assays. For all other gene pairs, we then compared the conditional genetic interaction score with the phenotypes observed in the conditional viability assays. The median conditional *z*-scores were found to be correlated with the qualitative ranking of the condition viability spot assays with median values of −3.64 for strong negative, −0.72 for negative, −0.014 for neutral, 0.94 for positive and 2.27 for strongly positive (Supplementary Fig S4). At the cut-off selected here, ∼70% (62/89) of the conditional genetic interactions showed a similar phenotype (positive, neutral or negative) as the viability assays. It is worth pointing out that visual inspection of spot tests is far less quantitative than *S*-scores derived from the genetic interaction screens. Nonetheless, the results above suggest that the static and conditional genetic interactions obtained are of high quality. The *S*-score and *z*-score values for gene–gene pairs in each condition are available in Supplementary Table S1.

### Specificity and functional relevance of conditional genetic interactions

For each of the conditions, we counted the number of conditional genetic interactions and the fraction of those that overlap with static genetic interactions in the same condition. Across all conditions, 32% of the conditional genetic interactions are observed in the corresponding static genetic interaction network. For the different conditions, this value ranges from 22% for CR and SO to 44% for one of the ZY experiments, which is similar to what was reported in a DNA damage differential E-MAP (38%) (Bandyopadhyay *et al*, 2010). This suggests that the static and differential interactions are mostly non-overlapping. In addition, the majority of differential interactions are not shared across stress conditions (Fig2B). We next studied the functional relevance and specificity of these two different networks by calculating the enrichment of known stress response genes. For each stress condition (except CR), we were able to compile a list of genes that, when mutated, confer sensitivity to the stress from unbiased genome-wide studies (Materials and Methods). We observed that pairs of these stress-sensitive genes are most often found to be significantly and specifically enriched in the corresponding differential network but not in the corresponding static network (Fig2C). Some of the observed cross-stress enrichment is expected. For example, the sorbitol-sensitive pairs are specifically enriched in the SO and also in the ZY differential network. This could be anticipated as ZY also activates the HOG pathway (Bermejo *et al*, 2008). Furthermore, since cell wall mutants are frequently caffeine sensitive, zymolyase-specific pairs are highly enriched in CA. Interestingly, hydrogen peroxide pairs are enriched under SO and ZY treatment, suggesting that members of the HOG and CWI pathways also collaborate in the response to oxidative stress. These results support previous reports that have related oxidative stress with these two pathways in yeast (Rep *et al*, 2001; Alic *et al*, 2003; Bilsland *et al*, 2004; Staleva *et al*, 2004; Petkova *et al*, 2012). We also observed that genes sensitive to rapamycin (a TORC1 complex inhibitor; Heitman *et al*, 1991) were specifically enriched in the caffeine differential network, reinforcing the idea that both drugs share cellular targets. Importantly, the enrichment of these known stress-related genes is much less significant and less specific in the static networks (Fig2C), showing the value of our approach for connecting the distinct signalling components with specific cellular stresses. These results suggest that genes that cause a conditional fitness defect when mutated are more likely to show conditional genetic interactions. To test this notion explicitly, we tested the correlation of single-mutant fitness (SMF) defect with the number of conditional genetic interactions for all array genes. We observed a significant but weak correlation between the absolute change in SMF and number of conditional interactions in SO (*r* = 0.11, *P*-value = 0.0003) and OX (*r* = 0.13, *P*-value = 4.52 × 10^−5^). These observations suggest that changes in SMF are significantly associated with the changes in genetic interactions in the same stress, but these account for a small fraction of the total variation observed.

### Degree of conditional interactions highlights key pathway-specific genes

We next asked whether the differential networks could be used to rank the query genes according to their involvement in the response to the distinct stresses. To this end, we ranked query genes according to the number of differential interactions in each condition (Fig2D). We note that only 12 query signalling genes were screened in both experiments (Fig1A, stared genes in Fig2D). The observed ordering recapitulates much of what was previously known about the protein members and the cellular functions of these pathways (Fig1B). The top-ranked query genes in SO include the MAPK and MAPKK of the HOG pathway, Hog1 and Pbs2, respectively, as well as Nmd5, a carrier protein required for the nuclear/cytoplasmic shuttling of Hog1 (Ferrigno *et al*, 1998). This suggests that the Hog1 nuclear translocation is important for full long-term response to osmostress. Nmd5 is in contrast absent from the list of top-ranked genes in ZY, consistent with the fact that Hog1 is not translocated to the nucleus after zymolyase-induced stress (Bermejo *et al*, 2008). The next top-ranked genes include the HOG pathway sensors Sho1 and Msb2 as well as Nbp2, an adaptor protein that targets the phosphatase Ptc1 to Pbs2/Hog1 to inactivate the pathway, suggesting the importance of Ptc1 for the modulation of the osmotic response. Among the top-ranked osmotic responding query genes is also the Ptk2 kinase. Although it has not been associated with the response to osmotic stress, Ptk2 is involved in the regulation of ion transport across the plasma membrane (Goossens *et al*, 2000). Our results underscore the relevance of this process in the long-term response to osmotic stress. Furthermore, Ptk2 has been shown to contribute to the osmotic stress response in *Neurospora crassa* (Lew & Kapishon, 2009). As expected, whereas kinases of the CWI and HOG pathway modules are in the top-ranked for ZY, only the ones belonging to the CWI cascade are in the top-ranked for CA. Under CA treatment, we find specifically members of the TORC1 (Tor1 and Kog1) but not TORC2 complex (Tor2 and Avo1). These results provide additional evidence on the functional connection between TORC1 and the CWI pathway (Yan *et al*, 2012). We also find Skn7 as the most responsive query gene under H_2_O_2_ in accordance with its role in the response to oxidative stress (Krems *et al*, 1996). Curiously the osmotic stress sensors Sho1 and Msb2 and the CWI kinases Pkc1 and Bck1 are also among the top-ranked genes in oxidative stress. Among them, only Pkc1 has been previously shown to be involved in the response to oxidative stress (Vilella *et al*, 2005). Although this work indicates that components downstream to Pkc1 in the CWI pathway do not participate in this response, our results suggest that Bck1 is relevant for the cellular response to this stress. For CR, we observed, as expected, members of the CWI pathway (Pkc1, Bck1 and Slt2) as well as the Tor2 and Ypk2 kinases. These results illustrate the close link between TORC2, the actin cytoskeleton regulation, and the CWI pathway. Furthermore, they also provide evidence that the homolog of mammalian kinase SGK Ypk2 is not only one of the TORC2 substrates (Kamada *et al*, 2005), but probably a major TORC2 downstream effector.

We compared the ranking of query genes obtained above with the fitness defect the same genes have when knocked out under the same conditions. For each of the query genes, we counted the number of differential interactions in SO, OX and CA and compared this value with the single-mutant fitness defect in appropriate conditions. We observed a significant correlation between the single-mutant fitness (SMF) defect and number of interactions in SO (*r* = 0.72, *N* = 28, *P*-value = 7 × 10^−6^) and OX (*r* = 0.52, *N* = 28, *P*-value = 0.0012) but not in CA (*r* = −0.04, *N* = 27). However, there is a correlation between the SMF defect in rapamycin and number of interactions in caffeine (*r* = 0.71, *N* = 24, *P*-value = 5 × 10^−5^). These observations lend credence to the notion that the degree of differential interactions associates signalling genes with their respective pathways. However, these correlations are driven by a small number of genes. For example, excluding *HOG1* and *PBS2* in SO, *SKN7* in OX and *TOR1* and *SLT2* in CA (versus rapamycin SMF) abolishes the correlations (*r* = −0.32, *r* = −0.26, *r* = 0.15, respectively). For the HOG pathway, we compared the ranking of known pathway members according to the number of differential interactions and SMF defect scores in osmotic stress. Some pathway members (*MSB2*, *NBP2* and *SHO1*) are among the top genes with most differential interactions but do not have a strong SMF defect. Similarly, *HOT1* and *SSK22* show a significant SMF defect but have fewer differential genetic interactions. Both the SMF and the degree of conditional interactions appear to provide complementary condition-specific functional information regarding single genes.

Together, the results on the specificity (Fig2C), reproducibility and functional ranking of query signalling genes (Fig2D) support the view that the differential genetic networks provide an accurate representation of the known functional interactions of these environmental response pathways. Furthermore, the unexpected observations constitute putative novel functional associations between signalling genes and the corresponding stress response.

### Condition-dependent gene–complex functional associations

**Figure 3:**
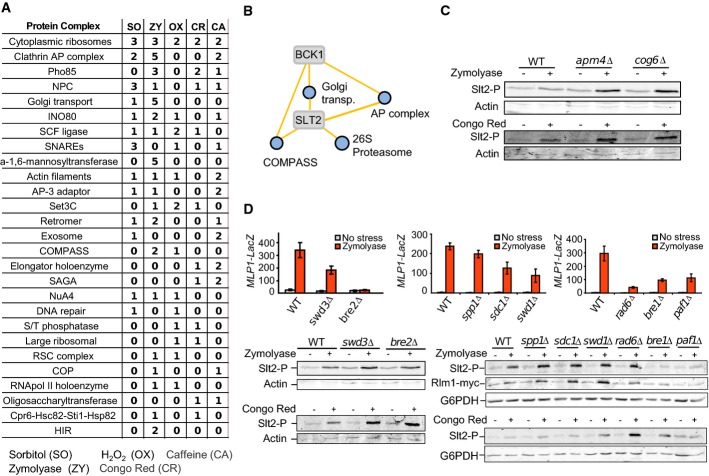
Putative gene–complex associations highlight novel roles for membrane trafficking and the COMPASS complexes in modulating the cell wall integrity pathway

Having established that the differential networks are enriched in known functional associations, we set out to identify previously undiscovered roles for protein complexes in the response to stress. For each protein complex represented in our array with at least four genes, we calculated the enrichment of differential interactions with each query gene in each stress condition (Materials and Methods). To summarize the results, we then summed the total number of significant gene–complex associations for each condition and ranked each complex according to these values (Fig3A). The most stress-responsive complex is the ribosome, likely due to the known importance of translational control in the cellular stress response (Holcik & Sonenberg, 2005). Several of the top-ranked complexes are related to membranes (e.g. Clathrin AP complexes, Golgi transport, SNAREs), which highlights the importance of membrane trafficking for the response to environmental changes. Genes responsible for membrane trafficking have also been shown to be a hub in the static genetic interaction network of growing cells (Costanzo *et al*, 2010). We also noted that the nuclear pore complex (NPC) has several functional associations with signalling genes in the presence of SO, suggesting a relevant involvement of this complex in response to osmotic stress. In fact, several nuclear pore proteins had been shown previously to have changes in phosphorylation after exposure to SO (Soufi *et al*, 2009). Hog1 has been shown to phosphorylate nucleoporins to control mRNA export upon stress, placing NPC as a clear HOG target for osmostress response (Regot *et al*, 2013).

Condition-specific associations between the query genes and protein complexes were predicted based on the enrichment of differential interactions. The total number of conditional gene–complex associations was calculated and complexes ranked based on the sum across all conditions.

Network diagram of the predicted zymolyase-specific gene–complex associations for Bck1 and Slt2 CWI kinases.

Western blotting analysis of Slt2 phosphorylation in WT (BY4741) and isogenic mutant cells lacking Amp4 (component of the AP complex) or lacking Cog6 (component of the COG complex). Cells were grown to mid-log phase at 24°C in YPD, and then, culture aliquots were treated or not with Congo red (30 μg/ml) or zymolyase 100T (0.8 U/ml) for 4 h. Proteins extracts were prepared, and phosphorylated Slt2 and actin (as a loading control) were detected with anti-phospho-p42/44 and anti-actin antibodies, respectively.

CWI pathway activity in WT (BY4741) and isogenic mutant strains lacking different components of the COMPASS, Paf1C and Rad6/Bre1 complexes. Top: Slt2- and Rlm1-dependent transcriptional induction of *MLP1*-lacZ was examined by β-galactosidase activity determination in cell extracts in the absence or presence of ZY. Aliquots of exponentially growing cultures of the distinct strains bearing the plasmid YEp352MLP1-lacZ were left untreated or treated with zymolyase 100T (0.8 U/ml) for 4 h, and β-galactosidase assays were performed. Data shown are the average of three independent experiments performed in triplicate. Error bars indicate standard deviations. Bottom: Western blotting analysis of cell extracts from exponentially growing cultures of the same strains as above, left untreated or treated with zymolyase 100T (0.8 U/ml) or Congo red (30 μg/ml) for 4 h. Phospho-Slt2, Rlm1-myc and actin or G6PDH (as a loading control) were detected with specific antibodies.

The membrane-related adaptor protein (AP) complexes, which coordinate cargo recruitment and clathrin assembly during clathrin-coated vesicle biogenesis (Yeung & Payne, 2001), show strong association with the CWI Slt2 and Bck1 kinases under the cell wall stress ZY (Fig3B). These results illustrate that not only exocytosis but also the endocytic process is important for cell wall homeostasis in conditions that alter the cell wall. Accordingly, deficiencies in these complexes would exacerbate the effects of ZY and trigger higher activity through the CWI pathway. In line with this prediction, we observed an increase in the phosphorylation of the CWI Slt2 kinase when the cargo-binding subunit of the AP-2 complex either Apm4 or Cog6 is removed and cells stressed with either ZY or CR (Fig3C). In fact, it has been recently described the role of Apm4 in the cell wall damage response, since the cell wall stress sensor Mid2 is a cargo for the AP-2 complex (Chapa-y-Lazo *et al*, 2014). It is interesting to note the retromer complex, which mediates sorting of retrograde cargo from the endosome to the trans-Golgi network (Attar & Cullen, 2010), also displays genetic interactions in the presence of ZY. We speculate that endocytic organelles also could play a direct role in yeast signalling modulation, as proposed for mammalian cells (Miaczynska *et al*, 2004).

The Slt2 and Bck1 kinases also show ZY-dependent associations with the histone H3 lysine 4 (H3K4) methylase COMPASS complex (Shilatifard, 2012) (Fig3B). Deletion of the COMPASS subunit-encoding genes *SWD3* or *BRE2* displays a significant reduction of the ZY-dependent induction of the *MLP1* promoter, a well-established reporter of the CWI transcriptional response (Rodriguez-Peña *et al*, 2008) albeit they show an increased Slt2 phosphorylation compared to the wild-type (Fig3D). A similar behaviour was observed in mutants affected in other components of COMPASS (*spp1*Δ, *sdc1*Δ and *swd1*Δ), suggesting that this complex participates in the Rlm1-regulated transcriptional response to cell wall stress (Fig3D). We also observed a decreased induction from the *CHR1* promoter (another reporter for the CWI pathway) in *bre2*Δ mutant cells (Supplementary Fig S5). The *bre2*Δ mutants do not show reduced induction of an osmostress-responsive promoter (*STL1*) or a mating pathway promoter (*FIG1*) under osmotic stress or in response to pheromones, respectively (Supplementary Fig S5).

Since the monoubiquitinase Rad6 is required for H3K4 trimethylation by COMPASS and Bre1 is in turn required for Rad6 recruitment to chromatin (Shilatifard, 2012), we next tested *MLP1* expression in mutants lacking components of this ubiquitin ligase complex. As observed in Fig3D, both Rad6 and Bre1 are required for ZY-induced *MLP1* expression. Strikingly, *bre1*Δ mutants do not show Slt2 phosphorylation and Rlm1 accumulation upon pathway stimulation, pointing to a role not only in transcription but in signal transmission to the MAPK. This result also suggests the importance of ubiquitination for post-transcriptional modulation of the pathway. We have also observed that the Paf1 complex (Paf1C), a platform for the recruitment of histone methyltransferases (Krogan *et al*, 2003), could also be participating in *MLP1* transcription, since *paf1*Δ also showed very reduced transcriptional induction of *MLP1-LacZ* (Fig3D). However, similar to *bre1*Δ, *paf1*Δ cells display low Slt2 phosphorylation and Rlm1 activation after ZY and CR stress (Fig3D), suggesting that this complex is also affecting signal transmission through the CWI pathway. Of interest, the Paf1 complex is necessary to prevent a defect in transcription elongation of the *FKS2* gene, a gene induced by an Rlm1-independent non-kinase-Slt2-dependent mechanism of transcription (Kim & Levin, 2011).

These results suggest that the differential interaction networks allow for an unbiased large-scale exploration of conditional dependent functional interaction networks. The full list of gene–complex conditional associations is provided in Supplementary Table S2.

### Conditional variation in genetic ‘finger prints’ recapitulates known condition-dependent associations

**Figure 4:**
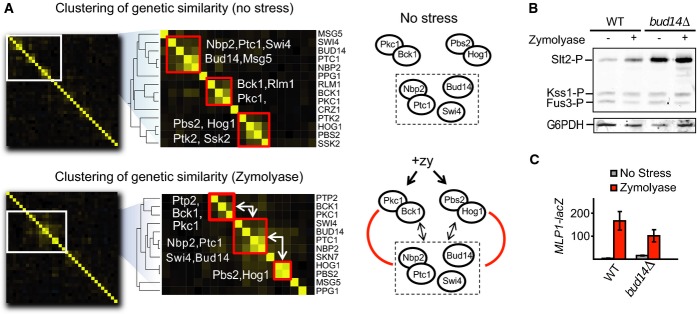
Correlation of genetic profiles under different conditions provides insights into condition-dependent functional interactions
A The vector of genetic interaction *S*-scores for each query gene was used to calculate pair-wise Pearson correlation coefficients for each condition. The matrix of correlations in the presence and absence of zymolyase was clustered and represented in the form of a heat-map. We highlighted the sections containing 3 clusters: Hog1/Pbs2 cluster; Pkc1/Bck1 cluster; and a cluster containing Nbp2, Ptc1 and Bud14. White arrows highlight the increase in average correlation of genetic-interaction scores across clusters in the presence of zymolyase.
B, C CWI pathway activity in WT (BY4741) and the isogenic *bud14*Δ strain, in the presence or absence of ZY. (B) Phospho-Slt2 levels and (C) transcriptional induction of *MLP1*-lacZ were analysed as in Fig3D and E. Phospho-Fus3 and Phospho-Kss1 are also detected with the anti-phospho-p42/44 antibody.

The vector of genetic interaction scores for each gene constitutes a functional ‘finger print’ that can be compared to highlight functionally related genes (Beltrao *et al*, 2010; Costanzo *et al*, 2010). Genes with highly correlated genetic interactions tend to be part of the same pathway/complex, and clustering of these scores has been shown to be a powerful way to identify novel pathway/complex members (Schuldiner *et al*, 2005; Collins *et al*, 2007; Ryan *et al*, 2012). We reasoned that condition-induced changes in correlation of genetic interactions scores could equally be used to identify conditional functional associations. For each pair of query signalling genes, we calculated the correlation of their genetic interaction scores in the presence and absence of stress. We then performed hierarchical clustering on the matrix of pair-wise correlations, and a heat-map representation of the clustering in the presence or absence of ZY is shown in Fig4A. As expected, regardless of the stress condition, the most correlated signalling genes tend to operate within the same pathway and very often their products physically interact (e.g. Nbp2-Ptc1, Pbs2-Hog1). In addition, we noted that, in the presence of stress, there are substantial changes in the clustering. In the presence of ZY, we noted in particular that a cluster containing Nbp2, Ptc1, Swi4 and Bud14 becomes more correlated with the Pbs2/Hog1 and Pkc1/Bck1 clusters (Fig4A). The changes in correlation for gene pairs between *NBP2/PTC1/BUD14* and *HOG1/PBS2* have a higher average increase under ZY than random gene pairs (0.12 versus −0.005, *P*-value = 0.0006; Wilcoxon rank test). The gene pairs between *PKC1/BCK1/PTP2* and *NPB2/PTC1/BUD14* also show a significant but marginal increase in average correlation in ZY compared to random pairs (0.04 versus −0.005, *P*-value = 0.04 Wilcoxon rank test). These changes are consistent with the well-characterized role of Nbp2-recruiting Ptc1 for the inactivation of Hog1 during adaptation (Mapes & Ota, 2004). Ptc1 and Nbp2 have also been shown to regulate Slt2 activity (Stanger *et al*, 2012). The similar behaviour between Bud14 and Nbp2/Ptc1 was not expected and suggests a role for Bud14 in either the HOG or the CWI pathways. Deleting *BUD14,* which codes for a regulatory subunit of the protein phosphatase type 1 Glc7, results in Slt2 hyper-phosphorylation even in the absence of stress (Fig4B), as it has been reported for *nbp2Δ* or *ptc1Δ* cells (González *et al*, 2006; Stanger *et al*, 2012). Thus, the CWI pathway activation could account for the close clustering of these mutants. Interestingly, in *bud14Δ* cells, the reduced transcriptional induction of *MLP1* does not correlate with the high MAPK phosphorylation (Fig4C), suggesting a role for Bud14 either in the appropriate signal transmission from Slt2 to the transcription factor Rlm1 or in the transcriptional process. No significant effect was observed either on the activity of the mating pathways as determined by Fus3 or Kss1 phosphorylation analysis (Fig4B) and *FIG1* transcription or on the activity of the *HOG* pathway, as measured by *STL1* transcriptional activation (Supplementary Fig S6).

Overall, the conditional changes in genetic interaction as measured by differential interaction scores or by changes in the correlation patterns are a powerful tool to identify condition-dependent functional interactions.

### Dissection of conditional genetic interactions using large-scale conditional regulatory data

**Figure 5:**
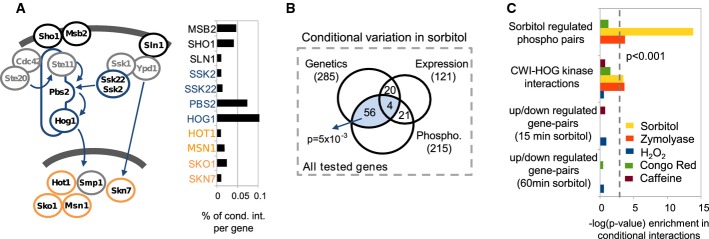
Dissection of differential genetic interactions into post-translational and transcriptional regulatory components

As we described above, differential genetic interactions are a measure that relate to the functional importance of pathway conditional interactions. This genetic information is highly complementary to other studies that attempt to dissect the post-translational or transcriptional mechanisms of stress response pathways. Previous studies have combined genetic and physical interaction data to study the interrelationships between pathways and complexes (Kelley & Ideker, 2005). We reasoned that combining differential genetic data with conditional phosphoproteomic and transcriptional information would allow us to dissect the relative importance of these different response mechanisms. To test this, we focused on the HOG pathway and compiled previously published phosphoproteomic (Soufi *et al*, 2009) and gene expression changes (Gasch *et al*, 2000) in the presence of SO. We first ordered the query signalling genes screened in our study according to their role in the information flow of the pathway (i.e. sensing, post-translational and transcriptional). We observed that the upstream sensing and post-translational regulators, in particular for the Sho1 branch of the pathway, explained more of the differential interactions than the downstream transcriptional regulators (Fig5A). From the set of approximately 1,200 genes tested in our array, we then looked at the overlap between those that had at least two differential interactions and those that showed changes in phosphorylation or changes in gene expression in the presence of SO (Fig5B). The genes with differential interactions overlapped significantly with the set of proteins that are regulated by phosphorylation (1.4 times above random, *P*-value = 0.0051 with a Fisher's exact test) but not with the set of differentially expressed genes (0.8 of random expectation, *P*-value = 0.08 for depletion with a Fisher's exact test). Finally, we tested the enrichment of different gene pairs in the differential networks: gene pairs that code for SO phospho-regulated proteins; gene pairs that code for known kinase–substrate interactions in the CWI or HOG pathways (obtained from phosphoGRID—www.phosphogrid.org); and gene pairs that are differentially expressed after exposure to SO. Only the gene pairs related to post-translational regulation were enriched in SO differential network (Fig5C). Kinase–substrate interactions are enriched fourfold (*P*-value = 3 × 10^−4^), while the phospho-regulated protein pairs are enriched twofold (*P*-value = 1 × 10^−14^). Overall, these results suggest that, for the HOG pathway, the sensing and post-translational regulation is of higher functional importance than the down-stream transcriptional response to stress. An alternative explanation for these observations would be that the post-translational regulatory response is more condition specific than the gene expression program. In fact, it has been shown that many stress conditions as well as many genetic perturbations result in a common change in the gene expression program that is commonly known as the environmental stress response (ESR) (Gasch *et al*, 2000). The ESR expression signature has been more recently linked to changes in the distribution of cells along the cell cycle caused by many stresses or genetic perturbations (Brauer *et al*, 2008; Duibhir *et al*, 2014). For this pathway, previous experimental evidence validates this prediction as it has been shown that the cell can grow under osmotic stress in the absence of a Hog1-dependent transcriptional response (Westfall *et al*, 2008). The changes in gene expression might be more relevant as a general stress response mechanism. We note that this might not be a general case for all pathways. Based on the fraction of differential interactions explained by each query gene (Fig2D), transcriptional related genes are highly ranked in the response to OX (Skn7) and CR (Swi4), but kinases dominate the top-ranked genes for the other stresses. These analyses suggest that differential genetic networks provide a way to dissect the functional relevance of different cellular response mechanisms.

Representation of the HOG pathway and the fraction of SO-dependent genetic interactions explained by different types of query genes: sensors (black), kinases (blue) and transcription factors (orange).

Venn diagram with the overlap between array genes that show at least two SO conditional genetic interactions, conditional changes in gene expression or conditional changes in phosphorylation.

Significance of enrichment of post-translational or transcriptionally regulated gene pairs in differential genetic interaction networks.

### Perspective

We provide here an extensive exploration of the differential genetic interactions for the environmental response MAPK pathways of *S. cerevisiae* using the dE-MAP approach. Differential genetic networks recapitulate many of the known condition-specific functional interactions and allowed us to predict novel functional associations. Conditional genetic interactions tend to have little overlap across conditions and show a small but significant correlation with the changes in single-mutant fitness under the same conditions. This is in line with the observation that gene knock-outs with slow growth under optimal conditions display more negative genetic interactions (Costanzo *et al*, 2010). Conditional single-mutant fitness information is less costly to collect than conditional genetic interactions so it can be used as an indicator for genes that are more likely to show changes in functional associations in a given condition. However, conditional single-mutant fitness information alone cannot identify the pairs of genes that show changes in functional associations under certain conditions, much like the growth phenotype of single-gene deletions under normal growth cannot identify specific pairs of genes that are synthetic lethal. We believe the data provided here will spur additional studies of such conditional functional and physical interactions.

Understanding the genetic make-up of an organism is important not just to discover novel functional associations but also to understand evolutionary constraints and the complexity of trait heritability. Studies in yeast suggest that genetic interactions contribute very significantly for quantitative trait heritability (Bloom *et al*, 2013). Therefore, genetic interactions across multiple loci are likely to be playing a significant role in the susceptibility to common human diseases. Given the lack of statistical power to identify genetic interactions of even the largest association studies, it becomes increasingly important to understand the general properties of the genetic architecture of the cell. While recent studies have made progress in predicting phenotypes from genome sequence (Jelier *et al*, 2011), we expect that static and differential genetic interactions will provide additional power for such predictions. It will also be increasingly important to understand what properties of the genetic architecture of the cell are conserved across species (Roguev *et al*, 2008; Ryan *et al*, 2012) and tissue types.

## Materials and Methods

### ‘Static’ and differential genetic interactions

Genetic interaction screens were performed as previously described (Collins *et al*, 2006) with the exception that the last selection step was done by replica-plating cells onto DM medium (no stress) and DM medium containing 0.6 M SO, 50 μg (1 U)/ml zymolyase 20T (MP Biomedicals Inc, Aurora, OH, USA) or 1.5 mM H2O2 (Sigma) for the first screen and 30 μg/ml Congo Red (Sigma), 4 mM caffeine (Sigma) or 50 μg (1 U)/ml zymolyase 20T for the second screen. For Congo red and zymolyase plates, the medium was buffered with 50 mM MES pH 6.5. Plates were incubated for 24/36 h at 30°C. Query mutant strains lacking genes *OPY2, MSB2, RLM1, HOT1, SKO1, NMD5, MSN1, MLP1, CHS3, SLM1* and *SLM2* were obtained as previously described by deleting the corresponding genes with the NAT^r^ gene, after PCR amplification from plasmid pFA6-NAT-MX6 (Goldstein & McCusker, 1999). Other query strains were previously described (Fiedler *et al*, 2009). The static genetic interactions (*S*-score) in each condition were scored as previously described (Collins *et al*, 2006). Values of |*S*-score| ≥ 3 were considered strong static genetic interactions. In order to identify genetic interactions that change significantly in the presence of a stress conditions, we calculated a *z*-score for each stress *S*-score (*S*_stress_) using an approach similar to that used by Bandyopadhyay and colleagues (Bandyopadhyay *et al*, 2010). As previously noted, the standard deviation of *S*-scores increases non-linearly with the magnitude (Bandyopadhyay *et al*, 2010). For this reason, the mean and standard deviation of the non-stressed *S*-score (*S*_control_) were calculated as non-parametric functions μ(*S*_control_) and σ(*S*_control_) using a sliding window. The differential *z*-score was calculated as (*S*_stress_ − μ(*S*_control_))/σ(*S*_control_). We used a threshold of |*z*-score| ≥ 2 to define the set of differential interactions in each condition. The *S*-score and *z*-score values for gene–gene pairs in each condition are available in Supplementary Table S1.

### Validation with spot assays

Qualitative ranking of cellular fitness in spot assays was performed with wild-type BY4741 or isogenic mutant cells. *SLT2* was deleted on selected single mutants included in the array (BY4741 background) with a PCR-amplified *SLT2* deletion cassette containing the *Kluyveromyces lactis URA3* marker. In this cassette, the marker is flanked by identical *Staphylococcus aureus* Sau96I DNA methyltransferase sequences. Following gene deletion, these sequences allow FOA selection of *URA3* popping-out cells. *hog1*Δ double mutants were constructed with a similar strategy or using a NAT^r^ deletion cassette.

### Enrichment of gene-sensitive pairs

Genes that result in sensitivity to stress, when knocked out, were compiled from previously genome-wide studies (curated from the SGD database—www.yeastgenome.org). We calculated the enrichment of pairs of sensitive genes in the static and differential genetic interactions, and significance was assessed using the hypergeometric distribution.

### Gene–complex associations

For each protein complex represented in our array with at least four genes, we calculated the enrichment of differential genetic interactions with each query signalling gene and each condition. Statistically significant signalling gene–complex associations were then selected for each condition. We summarized the results for each complex by summing the number of significant query–complex associations found in each condition. For this analysis, we used a larger number of differential interactions using a threshold of |*z*-score| ≥ 1.7. Enrichment significance was calculated using the hypergeometric distribution, and false discovery rates were determined by permutations as implemented in GO-TermFinder. A false discovery rate < 15% was used as the cut-off to select the gene–complex associations based on the differential genetic interactions. A list of gene–complex associations with significance values is provided in Supplementary Table S2.

### Plasmids

In order to determine *MLP1* transcriptional induction, the episomic vector YEp352 bearing a *MLP1*-GFP fusion (pMLP1-GFP) (Rodriguez-Peña *et al*, 2008) and the pMLP1-LacZ, carrying the transcriptional fusion of the *MLP1* promoter to the *lacZ* gene (García *et al*, 2009), were used. pCRH1-LacZ was also used to analyse *CRH1* transcriptional induction and therefore CWI activity (Bermejo *et al*, 2008). pRS315 backbone vectors carrying GFP under the control of the *STL1* (Wei *et al*, 2012) or *FIG1* promoters (Bashor *et al*, 2008) were used for analysis of the HOG and mating transcriptional induction, respectively.

### Immunoblot analysis of yeast cell extracts

Budding yeast extracts and Western blotting analysis of the distinct proteins were performed as previously described (Martin *et al*, 2000). Immunodetection of actin, Rlm1-Myc and glucose-6-phosphate dehydrogenase proteins was carried out using monoclonal C4 (Immuno MP Biomedicals, Catalog #: 69100), 9E10 (Santa Cruz Biotechnology, SC-40) and polyclonal anti G-6-PDH (Sigma, Product No. A 9521) antibodies, respectively. Monoclonal anti-phospho-p44/p42 MAPK (Thr202/Tyr204, Cell Signaling, mAb #: 4370) was used for detecting phospho-Slt2, phospho-Fus3 and phospho-Kss1. These primary antibodies were detected using a fluorescently conjugated secondary antibody from LI-COR Biosciences with an Odyssey Infrared Imaging System (LI-COR Biosciences).

### β-Galactosidase activity and flow cytometry assays

β-Galactosidase activities were determined according to Guarente (1983). Values are averages of at least three independent transformants assayed in triplicate. For GFP analysis, cells were collected, washed twice with PBS, treated with cycloheximide (10 μg/ml) and then analysed by flow cytometry in a Guava EasyCyte flow cytometer, acquiring green fluorescence through a 488 excitation laser and a 525/30 BP emission filter (BFP). The marker was set using unstained yeasts as controls.

## Supporting Information

Supplementary Legends

Review Process File
