# Supplementary figures and images for: Differential genetic interactions of yeast stress response MAPK pathways
Source: Mol Syst Biol. 2015 Apr 17;11(4):800. doi: 10.15252/msb.20145606 (PMC4422557; doi:10.15252/msb.20145606)

Un-stressed  
Biological replicates

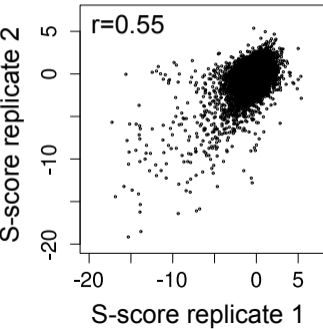

Zymolyase  
Biological replicates

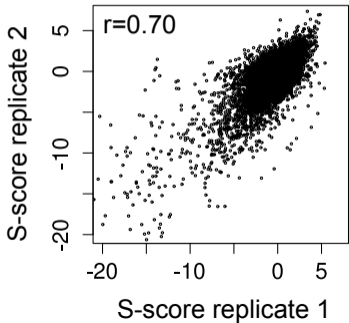

Supplement: Supplementary file 1 [file msb0011-0800-sd1.pdf]

Screen 1 z-score (ZY vs No-stress)

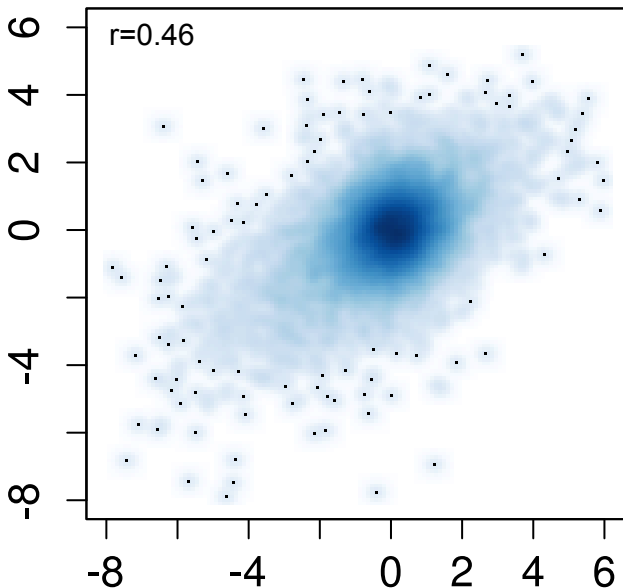

Screen 2 z-score (ZY vs No-stress)

Supplement: Supplementary file 2 [file msb0011-0800-sd2.pdf]

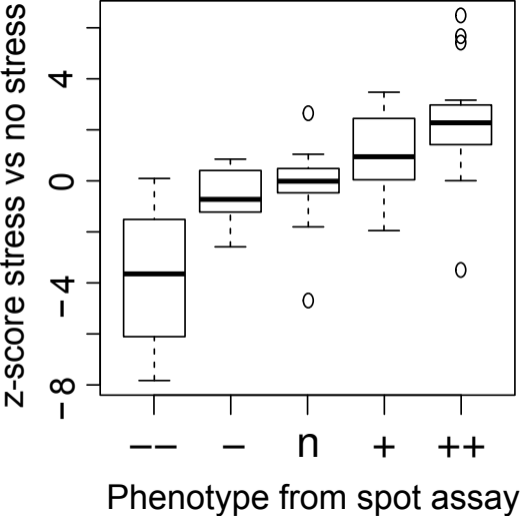

Supplement: Supplementary file 4 [file msb0011-0800-sd4.pdf]

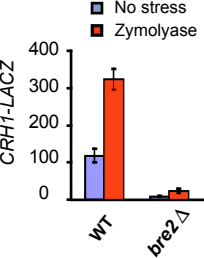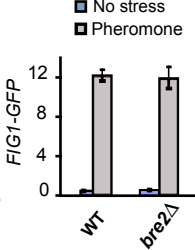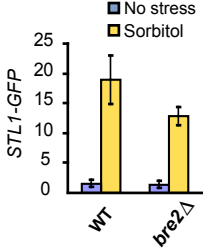

Supplement: Supplementary file 5 [file msb0011-0800-sd5.pdf]

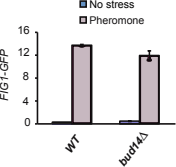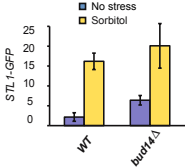

Supplement: Supplementary file 6 [file msb0011-0800-sd6.pdf]
