# Supplementary material for: Differential genetic interactions of yeast stress response MAPK pathways
Source: Mol Syst Biol. 2015 Apr 17;11(4):800. doi: 10.15252/msb.20145606 (PMC4422557; doi:10.15252/msb.20145606)
Supplement: Supplementary file 3 [file msb0011-0800-sd3.pdf]

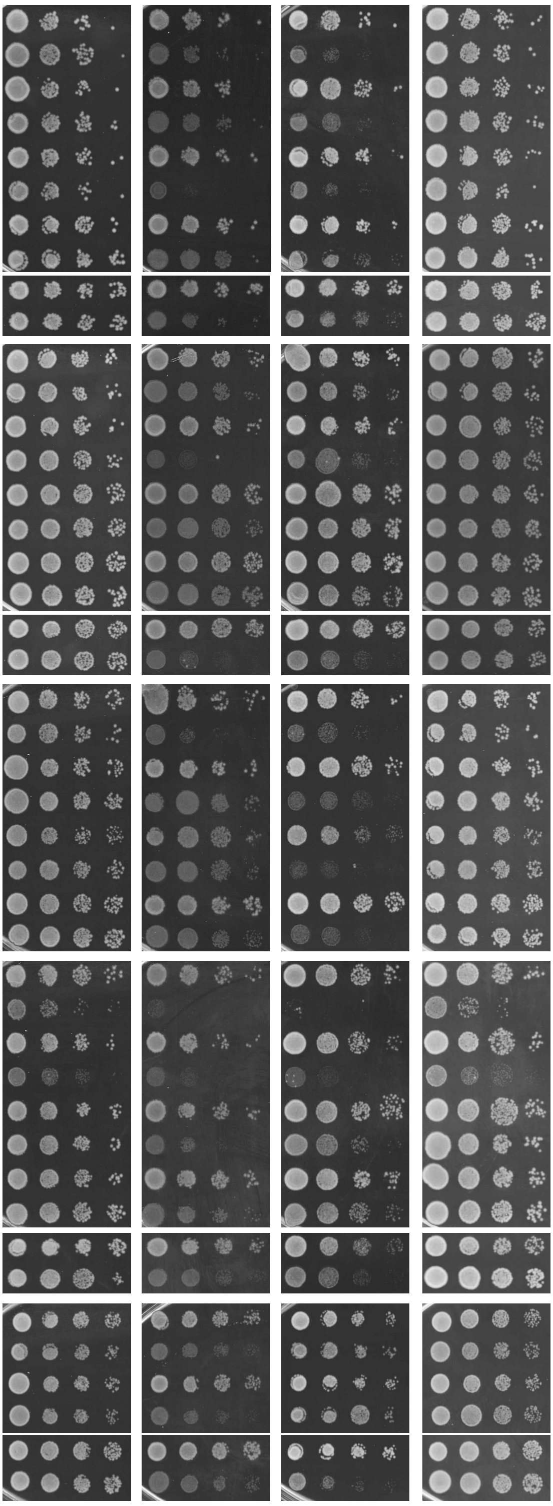

	SO		ZY		OX	
	Z-score	Drops	Z-score	Drops	Z-score	Drops
hog1Δ hst1Δ	0,042	+	-0,014	n	0,423	n
hog1Δ set3Δ	-0,191	-	-1,257	n	0,999	n
hog1Δ itc1Δ	2,046	++	1,193	++	-0,262	n
hog1Δ isw2Δ	2,651	n	1,645	++	-0,476	n
hog1Δ ppz1Δ	6,485	++	2,606	++	-1,05	n
hog1Δ msg5Δ	-7,827	- -	-0,082	n	-0,323	n
hog1Δ ste50Δ	5,672	++	1,961	++	-0,691	n
hog1Δ sko1Δ	5,422	++	2,784	++	0,208	n
hog1Δ vps72Δ	-2,133	- -	-1,95	+	0,018	n
hog1Δ nup2Δ	-1,223	-	-1,25	n	0,307	n
hog1Δ cue1Δ	3,164	++	-0,461	n	0,629	n
hog1Δ erg3Δ	0,005	++	0,683	n	0,313	n
hog1Δ ptc7Δ	2,246	++	1,042	n	0,001	n
hog1Δ pho80Δ	1,538	?	3,742	?	0,753	n
hog1Δ vps9Δ	1,025	?	-2,059	?	-0,032	n
hog1Δ pho23Δ	0,406	-	0,221	+	-0,18	n
hog1Δ sum1Δ	2,598	++	-3,493	++	-0,342	n
hog1Δ fab1Δ	0,945	+	0,379	+	-0,325	n
hog1Δ elp6Δ	2,294	+	0,858	++	0,182	n
hog1Δ elp4Δ	3,104	+	2,306	++	0,207	n
hog1Δ pus1Δ	1,512	+	0,542	n	-0,735	n

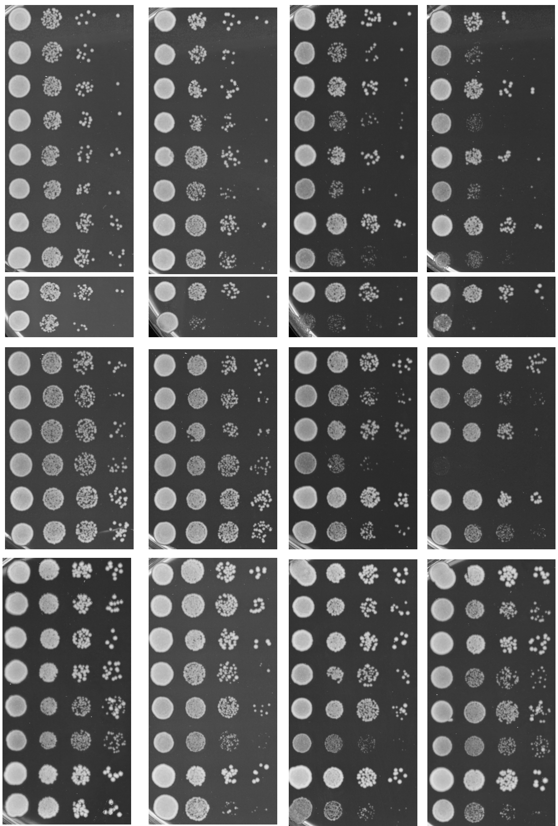

	CR		ZY		CA	
	Z-score	Drops	Z-score	Drops	Z-score	Drops
slt2Δ spp1Δ	-0,398	n	-2,152	-	-0,577	-
slt2Δ swd1Δ	1,002	n	0,470	-	0,550	n
slt2Δ get1Δ	-1,804	n	-1,174	- -	-5,967	- -
slt2Δ pho80Δ	0,093	- -	-0,265	- -	-6,247	- -
slt2Δ ssk2Δ	0,951	n	3,474	+	2,616	+
slt2Δ ubp3Δ	-0,627	n	-2,499	- -	-6,303	- -
slt2Δ slm1Δ	-1,420	+	2,446	+	-0,767	+
slt2Δ sdc1Δ	0,022	n	-1,395	n	-2,586	-
slt2Δ swd3Δ	-0,872	-	-4,045	- -	-4,691	n
slt2Δ cog6Δ	0,850	- -	-3,647	- -	-0,892	- -
