# Supplementary material for: Differential genetic interactions of yeast stress response MAPK pathways
Source: Mol Syst Biol. 2015 Apr 17;11(4):800. doi: 10.15252/msb.20145606 (PMC4422557; doi:10.15252/msb.20145606)
Supplement: Supplementary file 9 [file msb0011-0800-sd9.docx]

**Supplementary Table and Figure Legends**

Supplementary Table S1 – Genetic interaction S-scores and conditional genetic interaction z-scores for gene-gene pairs in each condition. For each gene-pair analysed in our screen we provide the static genetic interactions (S-score) in each condition and the calculated conditional genetic interaction z-score.

Supplementary Table S2 – Gene-complex associations. For each protein complex represented in our array with at least 4 genes we calculated the enrichment of differential genetic interactions with each query signalling gene and each condition. Statistically significant signalling gene-complex associations were then selected for each condition.

Supplementary Figure S1 – Correlation of genetic-interaction scores (S-score) for biological replicates. We correlated the S-scores obtained for the overlapping gene-pairs measured in both screens (figure 1) either in un-stressed conditions or in the presence of zymolyase. We observed correlation coefficients that are similar or better than obtained in previous genetic interaction screens.

Supplementary Figure S2 – Correlation of conditional genetic-interaction scores (Z-score) for biological replicates. We correlated the conditional genetic interaction z-scores obtained for the overlapping gene-pairs measured in both screens for the zymolyase condition.

Supplementary Figure S3 – Validation of conditional genetic interactions with spot assays. We tested the sensitivity of wild type (BY4741) and selected isogenic single and double mutants to different stresses. Ten-fold serial dilutions of cell suspensions were spotted onto YPD plates supplemented with the indicated drugs and incubated at 30°C for 42 h, excepting the lowest row of panels that were incubated for 56h. Sensitivity of *hog1Δ* mutants was analysed in plates containing 0.6 M sorbitol (SO), 50µg (1U) /ml) zymolyase 20T (ZY) and 3 mM hydrogen peroxide (OX). Sensitivity of *slt2Δ* mutants was analysed against 0,3µg/ml Congo red (CR), 10µg (0.2U)/ml zymolyase 20T (ZY) and 2 mM caffeine (CA). For comparison, the scores for genetic interaction under stress are also included from strongly conditional negative to strongly conditional positive (--/-/n/+/++), together with the Z-score for each specific gene pair and stress condition.

Supplementary Figure S4 – Comparison between conditional genetic interaction z-score with phenotypes from spot assays. For all pairs of double mutants tested in the spot assays we scored the change in genetic interaction in a qualitative schema with 5 groups from strongly conditional negative to neutral and to strongly conditional positive (--/-/n/+/++). For each of these groups we plotted the distribution of z-score for the corresponding conditional genetic interaction using boxplots. The median conditional z-scores are -3.64 for strong negative (--), -0.72 for negative (-), -0.014 for neutral (n), 0.94 for positive (+) and 2.27 for strongly positive (++).

Supplementary Figure S5 – Specificity of the *bre2Δ* transcriptional phenotype. The transcriptional output for CWI, mating and HOG pathways were tested using canonical promoter reporters (*CRH1*, *FIG1* and *SLT1* respectively). The activation of the *CRH1* transcriptional response was observed to be dependent on *BRE2*. The *bre2Δ* mutant had very little effects on the transcriptional induction of the mating and osmotic pathway reporters. WT (BY4741) and the isogenic *bre2Δ* mutant cells carrying the corresponding reporter plasmids were grown to mid-log phase at 24 °C in YPD and then culture aliquots were treated or not with zymolyase 100T (0,8U/ml) for 4 h, α-factor (3µM) or sorbitol 1M for two hours. Cells were collected and processed for β-galactosidase or flow cytometry analysis. Error bars represent standard deviation of three experiments.

Supplementary Figure S6 - Bud14 has no significant effect on signal transmission on mating and HOG pathways. WT (BY4741) and the isogenic *bud14Δ* mutant cells carrying the corresponding reporter plasmids were grown to mid-log phase at 24 °C in YPD and then culture aliquots were treated or not with α-factor (3µM) (a) or KCl 0,6M (b) for two hours. Cells were collected and processed for flow cytometry analysis. Error bars represent standard deviation of three experiments.
