# Supplementary material for: Differential genetic interactions of yeast stress response MAPK pathways
Source: Mol Syst Biol. 2015 Apr 17;11(4):800. doi: 10.15252/msb.20145606 (PMC4422557; doi:10.15252/msb.20145606)
Supplement: Supplementary file 10 [file msb0011-0800-sd10.pdf]

Differential genetic interactions of yeast stress response MAPK pathways

Humberto Martin, Pablo Ferndandez Pinar, Mike Shales, Ping Wei, Dorothea Fiedler, Kevan Shokat, Pedro Beltrao, Maria Molina, Wendell A. Lim, Nevan Krogan

Corresponding author: Pedro Beltrao, European Molecular Biology Laboratory - European Bioinformatics Institute

Review timeline:

Submission date:	22 July 2014
Editorial Decision:	26 August 2014
Revision received:	27 January 2015
Editorial Decision:	24 February 2015
Revision received:	16 March 2015
Accepted:	23 March 2015

Editor: Maria Polychronidou

Transaction Report:

(Note: With the exception of the correction of typographical or spelling errors that could be a source of ambiguity, letters and reports are not edited. The original formatting of letters and referee reports may not be reflected in this compilation.)

1st Editorial Decision 26 August 2014

Thank you again for submitting your work to Molecular Systems Biology. We have now heard back from the three referees who agreed to evaluate your work. As you will see from the reports below, the referees acknowledge that the presented conditional genetic-interaction data are potentially interesting. However, they raise a series of concerns, which should be carefully addressed in a revision of the manuscript.

Without repeating all the comments listed below, the more fundamental issues refer to the need to include further systematic analyses of the condition-specific genetic interactions and to investigate the parameters (i.e. interaction strength) potentially underlying the altered significance of the interactions between conditions. Moreover, the reviewers' mention that the methodology and statistical analyses should be described in better detail and that additional relevant references need to be included.

Reviewer #1:

The authors present an analysis of quantitative genetic interactions between ~50 query genes and ~1000 array genes in 5 conditions related to stress responses in yeast. The approach and analyses are very similar to a series of recent papers from the same lab but here are targeted at different query genes and conditions. Thus although the novelty is not high the data is interesting because there have been few conditional genetic interaction maps constructed.

© European Molecular Biology Organization

Abstract:
The abstract is vague and rather superficial - the authors should more accurately describe the scale of what was actually done (=~ 49 x ~1200 in five conditions) and the details of the results rather than e.g. simply stating 'a large scale genetic interaction analysis...' As a second example, the last line about the integration with other datasets describes what was done but not what the results or conclusion were.
'resulting in 250,000 condition specific interactions'. This is wrong. The authors tested ~250k possible interactions but most of these are scored as NOT interacting. Please change the number or text.

Intro
The authors have a tendency to over-emphasise the contributions of the Krogan lab. For example, 'We have previously shown that quantitative genetic interaction mapping can be used to study the functional organisation...[self citations]" is correct, but so have several other labs and papers! Slightly more modesty and balance would only reflect well on the authors!

Main text
'significant genetic interactions (here defined as abs(S-score) >= 3)" Please sate the FDR when using this score rather than simply writing 'significant' in the main text (either based on this data or on previous data produced using the same assay).

Throughout the text, every time a p-value is used to support a result, the effect size should also be given. e.g. in the comparison with phosphorylation and expression. P-values are not particularly useful alone when evaluating results form large-scale genomic comparisons with large N.

'static and differential are mostly non-overlapping...' This statement should be quantified by comparing to the overlap with two independently constructed static maps.

Expression analysis - in their analysis/discussion of the expression analysis, the authors should consider the known cell cycle/metabolic cycle response (Botstein, Holstege) which likely underlies much of the global expression response to stress in yeast (response previously referred to as the environmental stress response (ESR)).

Role of COMPASS. Chromatin complexes regulate the expression of many genes, alter diverse phenotypes and have been repeatedly identified as interacting promiscuously in genetic interaction screens. The authors should therefore be careful not to over-emphasise the insight from this result.

Reviewer #2:

Summary:
This study identifies yeast genetic interactions that are specific to stress conditions caused by sorbitol, hydrogen peroxide, zymolyase, Congo red, and caffeine. The authors have previously applied similar methods (calculating differential interactions) to study DNA damage E-MAP screens and this study is a meaningful continuation of interrogating yeast genetic interactions in stress environments. A small set of 49 stress-pathway-relevant queries was screened against an array of 1200 genes in the five stress conditions and a control condition and dE-MAPs were generated to identify condition-specific interactions. The authors show that the genes with many condition-specific interactions are reasonable given the conditions queried and discuss a number of specific examples where the differential interactions highlight biological relationships. This is a new dataset in the important area of differential network analysis. The study is of potential broad interest, but in its current form, the manuscript lacks depth in terms of systematic analysis and evaluation of the dataset (see specific examples below). I would also note that despite the importance of this area of work and the fact that new differential network data will be of interest to the community, the conceptual novelty of insights in the paper about how networks are rewired or general principles for analyzing this type of data are relatively low. The manuscript is relatively stronger in discussion of specific biological examples and insights that differential interactions bring to those examples. The ultimate impact of this paper will be due to the data presented in it, so it's important that authors present systematic evidence for its quality and utility.

Major comments:
(1) The main text does not discuss the fact that different queries are used for different environments. While this is clear from Figure 1, it should be explained in the main text to accurately represent the experiments performed. The last panel of Figure 2d also ignores this important point; a couple genes stand out, such as NMD5 and PTC1, for mainly having interactions from one screen, but two of the screens didn't include these queries. The authors should consider highlighting/starring genes that were queried in both screens.
(2) How many array genes are there? The authors first state 1200 (page 3), then claim 1300 (page 9) How were the array genes chosen? Does the choice of these genes affect the enrichments of literature-supported pairs? For example, if some array genes are functionally biased and some have unknown functions or low degree, the enrichments would be unsurprising.
(3) What's the overlap between static interactions in their study and those reported in the literature? The authors should provide an independent quality metric for their data before moving into interpretation of differential interactions.
(4) The systematic evaluation of the actual condition-specific genetic interactions reported seems weak. Most of the analyses focus on the condition-specific degree of genes and illustrate that genes that gain many interactions across conditions make sense given the condition. Enrichment for single mutant sensitivity in the corresponding conditions (as in Fig. 2C) provides some support but does not validate specific interactions. Readers' trust in the data could be significantly strengthened with more systematic evaluation of the specific interactions discovered.
(5) The method for scoring differential genetic interactions seems a bit ad hoc. Considering that there are published methods for this (e.g. Bean and Ideker Genome Biology 2012), the authors should either use the established method or demonstrate that their approach is superior.
(6) Page 6, 2nd paragraph: "..top ranked complexes related to membranes, clatherin AP complexes....highlights importance of membrane trafficking....". Membrane trafficking is a major hub in the static network so this does not seem to be specific to differential networks. The authors should comment on this.
(7) Can the authors comment on the overall difference of static vs differential interaction profile similarity networks? They focus on a small fraction of the network and highlight very modest differences. For example, they suggest that PCCs of 0.36 and 0.52 or 0.24-0.15 are different and biologically meaningful. Are these differences significant and meaningful? For example, even among members within the same protein complex under static conditions, this level of variation in the similarity might be observed in my experience.

Minor comments:
(1) Some of the figures are sloppily done. Fig 1b - there are question marks Figure 2a - The scatter plot is poorly made. Why are the static interactions that agree in the conditions partially removed and partially displayed? Figure 2d -the use of the word "explained" is confusing. Figure 3d uses "-" as a label.
(2) The manuscript needs proofreading. For example, there are missing words, extra words, typos, and the important term "genetic interaction" is repeatedly hyphenated.
(3) If the legend entries of Figure 1c were arranged in the same order as the bars, the labels could be understood on a black-and-white printout.
(4) The authors might consider color-coding the genes involved in different pathways in Figure 2d to better visualize results discussed in the text.

Reviewer #3:

The manuscript of Martin et al. provides genetic interaction (GI) maps across five different stress conditions to reveal condition-dependent functional interactions in yeast MAPK pathways. The study employs the well-established epistatic miniarray protocol and screens interactions between 49 signaling related query and 1200 array knockouts. Fitness was measured both under optimal growth conditions in the presence of five different stressors that act as signals for MAPK pathways. Based on these data, the authors generated a GI map for each condition (static maps) and also constructed differential interaction maps that reflect rewiring of interactions from unstressed to stress conditions. They used these maps to i) functionally rank query genes, ii) identify stress responsive protein complexes and perform in-depth analyses on some of them, iii) identify conditional functional

associations based on GI profile correlations and iv) show that rewiring of GI in the presence of sorbitol is correlated with changes in phosphorylation.
While the environmental-dependent rewiring of genetic interactions has been reported before, this study is unique in that it compares multiple stress conditions and uses this information to gain new insights into the functional interactions between MAPK pathway genes. Overall, this is an important contribution to MSB and will be of interest to yeast biologists, geneticists and functional genomicists. Nevertheless, I feel that addressing the following comments would further improve the paper.

Major points:
1) In general, a change in S-score (a measure of GI applied in this study) between conditions might arise for numerous different reasons: i) change in the strength of epistasis without a change in single mutant fitness (SMF), ii) change in the overall importance of the underlying biological process: while both SMF, DMF (double mutant fitness) and S-score change, the scaled epistasis value (ala Segre et al. Nat Genet 2004) might be not altered at all, iii) because S-score is not simply a measure of the strength of epistasis but also a measure of confidence (i.e. a z-score like index), it could change between conditions even if the absolute strength of epistasis is unchanged. Unfortunately, no analysis is presented to show which of the above might dominate the data. I have the feeling that change in the overall importance of the pathways without any specific change in the strength of the scaled epistasis might underlie much of the variation seen across stress conditions. If so, this would affect how we interpret the high prevalence of GI rewiring and could explain why the majority of differential interactions are not shared across conditions (page 4).

2) On page 5, the authors use the differential GI maps to rank genes according to their involvement in the different stress responses. While this approach recapitulates the known functional associations between signaling genes and specific stress responses, one might wonder how much of this information would have been provided by single mutant fitness data alone? Clearly, SMF should also change across conditions, and it remains unclear how much more information is provided by measuring double mutant fitness as well. It would be important to convince the readers that measuring GI maps across conditions captures much better condition-dependent functions than simply performing chemogenomic screens (which requires way less effort). In addition, the benchmark of the functional ranking of query genes is anecdotal and no statistics is provided.

Minor points:
1) Some relevant citations have been ignored, most notably the Costanzo et al. Science 2010 paper on the use of GI profiles as a measure of functional relatedness. Another example is Harrison et al. PNAS 2007 paper on the condition-dependency of GI in metabolism.

2) The observation that genes with differential interactions did not overlap with genes that are differently expressed could be linked to the finding that up/downregulation of a gene is usually not correlated with its change in importance (see Giaever 2002 Nature, Price et al. MSB 2013)

3) Fig 3A: color coding is confusing. Zymolase seems to be yellow / orange, not red as indicated in the legend. See also caffeine.

4) page 10, methods of identifying gene-complex associations. The method is not very clearly presented, I had to read the caption of Fig 3 to fully grasp it. Also, it's confusing why GO-TermFinder was used here - I guess it was for calculating the stats, not to perform GO analyses. The statistical test employed should be explicitly stated.

1st Revision - authors' response 27 January 2015

We thank the reviewers for their comments on our manuscript and we have addressed them accordingly in the text below. We hope that you will now find our revised acceptable for publication in Molecular Systems Biology. Reviewer comments are in black and our reply in blue.

Reviewer #1:
The authors present an analysis of quantitative genetic interactions between ~50 query genes and ~1000 array genes in 5 conditions related to stress responses in yeast. The approach and analyses are very similar to a series of recent papers from the same lab but here are targeted at different query genes and conditions. Thus although the novelty is not high the data is interesting because there have been few conditional genetic interaction maps constructed.

Abstract:
The abstract is vague and rather superficial - the authors should more accurately describe the scale of what was actually done (=~ 49 x ~1200 in five conditions) and the details of the results rather than e.g. simply stating 'a large scale genetic interaction analysis...' As a second example, the last line about the integration with other datasets describes what was done but not what the results or conclusion were.
'resulting in 250,000 condition specific interactions'. This is wrong. The authors tested ~250k possible interactions but most of these are scored as NOT interacting. Please change the number or text.

We have revised the abstract to address this concern raised by the reviewer. We have performed two separate experiments (32 x 1172 and 29 x 1185) under normal growth and 3 stress conditions each, where one of the stress conditions was common to both screen. Explaining the two experiments, their conditions and overlaps in the abstract would be overly verbose. Instead we have added to the abstract an approximate number of genetic crosses as suggested by the reviewer. It now reads: "*In this study, we have performed a large-scale genetic interaction analysis in S. cerevisiae of approximately 49 x 1200 double mutants in the presence of five different stress conditions, including osmotic, oxidative and cell-wall altering stresses*". We have also changed the abstract to reflect that 250,000 was the approximate number of measurements made not the number of significant conditional interactions and we now explain the results obtained from comparing the osmotic differential genetic interactions with the transcriptional and post-translational changes.

Intro
The authors have a tendency to over-emphasise the contributions of the Krogan lab. For example, 'We have previously shown that quantitative genetic interaction mapping can be used to study the functional organisation...[self citations]" is correct, but so have several other labs and papers! Slightly more modesty and balance would only reflect well on the authors!

We apologise for not referring sufficiently to previous literature related to quantitative genetic interaction screening. We have revised the introduction to address this concern.

Main text
'significant genetic interactions (here defined as abs(S-score) >= 3)" Please sate the FDR when using this score rather than simply writing 'significant' in the main text (either based on this data or on previous data produced using the same assay).

We acknowledge that we should have been more explicit about data quality metrics for the genetic interactions. Previous analysis of the accuracy of the S-score metric have shown that at the threshold used here (abs(S-score) >= 3) on the order of 80% of genetic interactions are genuine interactions (Collins et al. 2006). In order to further provide an estimate of accuracy for detection of genetic interactions at this cut-off we have compared biological replicates for two conditions (no-stress and zymolyase) for the double mutants that were screened in both conditions. For the normal growth condition, 53% and 55% of genetic interactions identified in screen 1 and screen 2 respectively are detected at the same or higher threshold in the replicate. For the zymolyase condition 51% and 63%

of genetic interactions revealed in screen 1 and screen 2 respectively are detected at the same or higher threshold in the replicate. We now describe these benchmarks in the main text.

Throughout the text, every time a p-value is used to support a result, the effect size should also be given. e.g. in the comparison with phosphorylation and expression. P-values are not particularly useful alone when evaluating results form large-scale genomic comparisons with large N.

In line with the reviewers' concern we have added effect size metrics wherever we provide p-values. Regarding specifically the comparison between the differential genetics and differential expression and phosphorylation the text now reads:
"The genes with differential interactions overlapped significantly with the set of proteins that are regulated by phosphorylation (1.4 times above random, p-value=0.0051 with a Fisher's exact test) but not with the set of differentially expressed genes (0.8 of random expectation, p-value=0.08 for depletion with a Fisher's exact test)." and "Only the gene-pairs related to post-translational regulation were enriched in SO differential network (Figure 5c). Kinase-substrate interactions are enriched 4 fold (p-value=3x10-4) while the phospho-regulated protein pairs are enriched 2 fold (p-value=1x10-14)."

'static and differential are mostly non-overlapping...' This statement should be quantified by comparing to the overlap with two independently constructed static maps.

We agree with the reviewer that this statement should have been quantified. For each of the conditions, we counted the number of conditional genetic interactions (abs(z-score)>2) and the fraction of those that overlap with static genetic interactions (abs(S-score)>3). Across all conditions 32% of the conditional genetic interactions are observed in static genetic interactions under the same condition. This is similar to the 38% overlap found in a DNA damage differential E-MAP (Bandyopadhyay et al. 2010). For the different conditions, this value ranges from 22% for Congo red and sorbitol to 44% for one of the zymolyase experiments. In addition, we have also compared the overlap of static and conditional genetic interactions across two independent experiments in zymolyase. For gene pairs for which we have static and zymolyase conditional genetic interactions in both screens we calculated the overlap between static and conditional across the replicates. For these pairs, 30% of conditional interactions from screen 2 overlap with the static genetic interactions in screen 1. Similarly, 39% of conditional interactions from screen 1 overlap with the static genetic interactions in screen 2. These values are in-line with the overlaps observed within each screen. We acknowledge that the initial statement was a miss-representation of the extent of overlap and thank the reviewer for this remark. We now provide this information in the main text.

Expression analysis - in their analysis/discussion of the expression analysis, the authors should consider the known cell cycle/metabolic cycle response (Botstein, Holstege) which likely underlies much of the global expression response to stress in yeast (response previously referred to as the environmental stress response (ESR)).

We agree with the reviewer that one explanation for the lack of overlap between the conditional genetic interactions and changes in gene expression under sorbitol could be partly due to what is commonly referred to as the environmental stress response. We had previously discussed this notion less explicitly: "Overall, these results suggest that, for the HOG pathway, the sensing and post-translational regulation is of higher functional importance <u>or is more condition-specific than the down-stream transcriptional response to stress</u>".
We now include a discussion regarding the general stress response in yeast as a possible reason for the potential lower specificity of the gene expression response.

Role of COMPASS. Chromatin complexes regulate the expression of many genes, alter diverse phenotypes and have been repeatedly identified as interacting promiscuously in genetic interaction screens. The authors should therefore be careful not to over-emphasise the insight from this result.

Although we agree with the reviewer that COMPASS is indeed very pleotropic, we provide several experimental results supporting a role for this complex in the regulation of the expression of 2 canonical promoters of the cell-wall integrity pathway under cell-wall stress. We have also shown

© European Molecular Biology Organization

that COMPASS does not appear to play a role in the induction of an osmostress promoter (*STL1*) or a mating pathway promoter (*FIG1*) under osmostress or in response to mating pheromone. We have described these results and argue we are not over-interpreting them in the manuscript.
In line with the reviewer's suggestion we made a small correction where we said before: *"This bre2Δ phenotype is specific to this pathway since the induction of an osmostress promoter (STL1) or a mating pathway promoter (FIG1) were not affected under osmostress or in response to pheromones respectively (supplementary figure 5)."* it now reads: "*The bre2Δ mutants do not cause an induction of an osmostress promoter (STL1) or a mating pathway promoter (FIG1) under osmostress or in response to pheromones respectively (supplementary figure 5)".*

Reviewer #2:

Summary:
This study identifies yeast genetic interactions that are specific to stress conditions caused by sorbitol, hydrogen peroxide, zymolyase, Congo red, and caffeine. The authors have previously applied similar methods (calculating differential interactions) to study DNA damage E-MAP screens and this study is a meaningful continuation of interrogating yeast genetic interactions in stress environments. A small set of 49 stress-pathway-relevant queries was screened against an array of 1200 genes in the five stress conditions and a control condition and dE-MAPs were generated to identify condition-specific interactions. The authors show that the genes with many condition-specific interactions are reasonable given the conditions queried and discuss a number of specific examples where the differential interactions highlight biological relationships. This is a new dataset in the important area of differential network analysis. The study is of potential broad interest, but in its current form, the manuscript lacks depth in terms of systematic analysis and evaluation of the dataset (see specific examples below). I would also note that despite the importance of this area of work and the fact that new differential network data will be of interest to the community, the conceptual novelty of insights in the paper about how networks are rewired or general principles for analyzing this type of data are relatively low. The manuscript is relatively stronger in discussion of specific biological examples and insights that differential interactions bring to those examples. The ultimate impact of this paper will be due to the data presented in it, so it's important that authors present systematic evidence for its quality and utility.

Major comments:
(1) The main text does not discuss the fact that different queries are used for different environments. While this is clear from Figure 1, it should be explained in the main text to accurately represent the experiments performed. The last panel of Figure 2d also ignores this important point; a couple genes stand out, such as NMD5 and PTC1, for mainly having interactions from one screen, but two of the screens didn't include these queries. The authors should consider highlighting/starring genes that were queried in both screens.

We agree with the reviewer and apologize for this oversight. We have changed the figure to highlight which strains were screened in both screens. We also describe this in more detail in the main text.

(2) How many array genes are there? The authors first state 1200 (page 3), then claim 1300 (page 9) How were the array genes chosen? Does the choice of these genes affect the enrichments of literature-supported pairs? For example, if some array genes are functionally biased and some have unknown functions or low degree, the enrichments would be unsurprising.

There are approximately 1200 genes that were measured in the array. The total number of genes that have at least one quantified genetic interaction is 1172 for screen 1 and 1185 for screen 2. We have corrected the error on page 9 (current page 12). This set of genes was selected to broadly cover different functional classes and it is similar to the array used in previous studies (Collins et al. 2007; Braberg et al. 2013). The enrichment analysis shown in Figure 2C was meant to serve as a benchmark for the conditional genetic interactions. We compared the enrichment of gene-pairs known to be important for the different responses in the static network versus the conditional-genetic interaction network. The same genes are present in the both types of network but they simply have different scores. Any bias in the array genes would be equally relevant to both the static

and conditional networks. For this reason, we consider that the enrichment of functionally related gene pairs in the conditional network versus the static network is not biased by the composition of the array

(3) What's the overlap between static interactions in their study and those reported in the literature? The authors should provide an independent quality metric for their data before moving into interpretation of differential interactions.

We agree with the reviewer that we should have provided additional quality metrics for the static (and conditional) genetic interactions reported. In order to further provide an estimate of accuracy for detection of genetic interactions at this cut-off, we have compared biological replicates for two conditions (no-stress and zymolyase) for the double mutants that were screened in both conditions. For the normal growth condition, 53% and 55% of genetic interactions called in screen 1 and screen 2 respectively are detected at the same or higher threshold in the replicate. For the zymolyase condition 51% and 63% of genetic interactions called in screen 1 and screen 2 respectively are detected at the same or higher threshold in the replicate. We have also compared the genetic interactions reported here with a previous study that contained some of the same gene-pairs (Fiedler et al. 2009). For the normal growth condition, 33% and 42% of genetic interactions called in screen 1 and screen 2 respectively were also in the previous study. These results are in-line with previous comparisons of genetic interaction screens (Ryan et al. 2012). We have now described these results in the main text.

(4) The systematic evaluation of the actual condition-specific genetic interactions reported seems weak. Most of the analyses focus on the condition-specific degree of genes and illustrate that genes that gain many interactions across conditions make sense given the condition. Enrichment for single mutant sensitivity in the corresponding conditions (as in Fig. 2C) provides some support but does not validate specific interactions. Readers' trust in the data could be significantly strengthened with more systematic evaluation of the specific interactions discovered.

We agree with the reviewer on this point and have invested a considerable effort into evaluating the quality of the reported conditional genetic interactions reported here. We have done additional computational as well as experimental validations.
For the zymolyase condition, we have conditional genetic interactions reported in the two biological replicates for a subset of all gene-pairs. For the biological replicates the correlation of the conditional genetic interaction scores is 0.46 (shown now in supplementary figure 2). We have also tested the reproducibility for the threshold selected. For this condition, 44% and 36% of conditional genetic interactions called in screen 1 and screen 2 respectively are detected at the same or higher threshold in the replicate
In order to validate the conditional genetic interactions experimentally, we have re-generated a set of 31 double-mutants, including 21 pairs with *HOG1* and 10 with *SLT2*. We compared the viability of the single mutants and double mutants in stress and no stress using spot assays (shown in supplementary figure 3). A total of 93 conditional genetic interactions were re-tested in this way. For all but 2 of the 31 pairs, the double-mutant did not appear to have a genetic interaction in absence of stress which is consistent with the no-stress S-score value for 90% of the pairs. We scored the change in genetic interaction in a qualitative schema with 5 groups from strongly conditional negative to strongly conditional positive (--/-/n/+/++). Two gene pairs showed negative-interactions in the absence of stress for the spot assays (hog1-pho80 and hog1-vps9). For these we could not easily score the change in genetic interaction in sorbitol or zymolyase in the spot assays. For all other gene-pairs we then compared the conditional genetic interaction score with the phenotypes observed in the conditional viability assays. The median conditional z-scores were found to be correlated with the qualitative ranking of the condition viability spot assays with median values of -3.64 for strong negative, -0.72 for negative, -0.014 for neutral, 0.94 for positive and 2.27 for strongly positive (shown in supplementary figure 4). Using the cut-off defined in the manuscript (abs(z-score)>2), 69,6% (62/89) of the tested conditional genetic interactions showed a similar phenotype (positive, neutral or negative) as the viability assays. We now describe these results in the main text.
The added work, in particular the experimental validation of the conditional genetic interactions, improves significantly the value of the dataset provided here. We think that the dataset provided which includes genetic interaction scores in different conditions and scores for the changes are of high quality.

(5) The method for scoring differential genetic interactions seems a bit ad hoc. Considering that there are published methods for this (e.g. Bean and Ideker Genome Biology 2012), the authors should either use the established method or demonstrate that their approach is superior.

The description of the method may not have been very clear. In fact, the approach is essentially the same as used in Bandyopadhyay *et al.* (Bandyopadhyay et al. 2010). As was the case for that article, we model the average difference in S-scores across two experiments as a function of the magnitude of the S-scores (e.g. the null-model). As described in supplementary materials (Bandyopadhyay et al. 2010), this was done in both cases using a sliding window and in both cases this null-model is used to define the significance of a conditional genetic interaction. The main difference between the two approaches was that Bandyopadhyay used two un-stressed replicates to model the null-distribution of S-score changes as a function of the S-score magnitude. In our case, we used directly the pair of stress and no stress conditions, under the assumption that conditional genetic interactions are rare. In our screen design the double-mutants were pinned in the different conditions (+/- stress) at the last selection step in order to reduce experimental variation. However, for this reason, we cannot use S-scores from two un-stressed conditions obtained from two independent screens to model the null-distribution of S-score differences. We could have included two separate un-stressed controls in each of the two screens but that would have resulted in 2 fewer conditions tested. We think the assumption that conditional genetic interactions are rare is a valid assumption. Any differences in the null-distribution introduced by using a pair of stress and no-stress conditions instead of two un-stressed conditions will be in the direction of under-estimating a significant conditional interaction. We have tried to emphasize in the fact that the method is similar to that used by Bandyopadhyay *et al.*

(6) Page 6, 2nd paragraph: "..top ranked complexes related to membranes, clatherin AP complexes....highlights importance of membrane trafficking....". Membrane trafficking is a major hub in the static network so this does not seem to be specific to differential networks. The authors should comment on this.

We agree with the reviewers comment and have added references supporting the observation that membrane trafficking also has many genetic interactions in static networks.

(7) Can the authors comment on the overall difference of static vs differential interaction profile similarity networks? They focus on a small fraction of the network and highlight very modest differences. For example, they suggest that PCCs of 0.36 and 0.52 or 0.24-0.15 are different and biologically meaningful. Are these differences significant and meaningful? For example, even among members within the same protein complex under static conditions, this level of variation in the similarity might be observed in my experience.

The reviewer is correct that we could have been more systematic about this analysis. To address this, we have calculated the changes in correlations for all double-mutants tested in zymolyase. We compared the changes in correlation for gene-pairs between the clusters with the changes occurring in all gene-pairs. The gene-pairs between *NBP2/PTC1/BUD14* and *HOG1/PBS2* have a higher average increase in correlation under zymolyase than random gene-pairs (0.12 versus -0.005, p-value = 0.0006 Wilcoxon rank test). The gene-pairs between *PKC1/BCK1/PTP2* and *NPB2/PTC1/BUD14* show a significant but marginal increase in average correlation in zymolyase compared to random pairs (0.04 versus -0.005, p-value = 0.04 Wilcoxon rank test). These results indicate that the changes in correlation between these clusters are significant. We have added these statistics to the appropriate section of the manuscript. More broadly, we currently lack systematic studies of changes of physical interactions of different types. Such studies would allow us to benchmark the biological significant of changes in correlations. We think that the availability of these data will also be an added incentive for such studies.

Minor comments:
(1) Some of the figures are sloppily done. Fig 1b - there are question marks Figure 2a - The scatter plot is poorly made. Why are the static interactions that agree in the conditions partially removed

and partially displayed? Figure 2d -the use of the word "explained" is confusing. Figure 3d uses "-" as a label.
(2) The manuscript needs proofreading. For example, there are missing words, extra words, typos, and the important term "genetic interaction" is repeatedly hyphenated.
(3) If the legend entries of Figure 1c were arranged in the same order as the bars, the labels could be understood on a black-and-white printout.
(4) The authors might consider color-coding the genes involved in different pathways in Figure 2d to better visualize results discussed in the text.

We appreciate the reviewer's attention to detail. We note that the question marks were not a mistake but meant to reflect our lack of knowledge regarding the pathway response driven by oxidative stress. We agree that it can be confusing and have removed them.
Regarding the scatter plot in Figure 2a we used 2 rectangles to emphasise the cut-offs used to select the static genetic interactions (abs(S-score)>3). We then coloured the conditional genetic interactions either in red or green. We are not sure what the reviewer means with the question "Why are the static interactions that agree in the conditions partially removed and partially displayed?". The interactions that we are highlighting are the conditional ones. We have modified the figure to make it clearer. We now have dotted lines indicating the thresholds used to define the static networks and a label to indicate which interactions were defined as conditional in this specific example.
We have carried out all suggestions made above except point number 4 since many query genes could be considered to take part in the response to different types of stress so it would be difficult to colour code them. We have instead highlighted the query genes that were analysed in both screens as per suggestion of another reviewer.

Reviewer #3:

The manuscript of Martin et al. provides genetic interaction (GI) maps across five different stress conditions to reveal condition-dependent functional interactions in yeast MAPK pathways. The study employs the well-established epistatic miniarray protocol and screens interactions between 49 signaling related query and 1200 array knockouts. Fitness was measured both under optimal growth conditions in the presence of five different stressors that act as signals for MAPK pathways. Based on these data, the authors generated a GI map for each condition (static maps) and also constructed differential interaction maps that reflect rewiring of interactions from unstressed to stress conditions. They used these maps to i) functionally rank query genes, ii) identify stress responsive protein complexes and perform in-depth analyses on some of them, iii) identify conditional functional associations based on GI profile correlations and iv) show that rewiring of GI in the presence of sorbitol is correlated with changes in phosphorylation.
While the environmental-dependent rewiring of genetic interactions has been reported before, this study is unique in that it compares multiple stress conditions and uses this information to gain new insights into the functional interactions between MAPK pathway genes. Overall, this is an important contribution to MSB and will be of interest to yeast biologists, geneticists and functional genomicists. Nevertheless, I feel that addressing the following comments would further improve the paper.

Major points:
1) In general, a change in S-score (a measure of GI applied in this study) between conditions might arise for numerous different reasons: i) change in the strength of epistasis without a change in single mutant fitness (SMF), ii) change in the overall importance of the underlying biological process: while both SMF, DMF (double mutant fitness) and S-score change, the scaled epistasis value (ala Segre et al. Nat Genet 2004) might be not altered at all, iii) because S-score is not simply a measure of the strength of epistasis but also a measure of confidence (i.e. a z-score like index), it could change between conditions even if the absolute strength of epistasis is unchanged. Unfortunately, no analysis is presented to show which of the above might dominate the data. I have the feeling that change in the overall importance of the pathways without any specific change in the strength of the scaled epistasis might underlie much of the variation seen across stress conditions. If so, this

would affect how we interpret the high prevalence of GI rewiring and could explain why the majority of differential interactions are not shared across conditions (page 4).

The reviewer raises here an important question regarding the underlying reasons behind the changes in S-score. To address this, we first tried to measure the extent by which the single mutant fitness (SMF) changes determine or correlate with changes in S-score across conditions. For the sorbitol and oxidative stress conditions we used previously reported SMF differences and correlated the absolute change in genetic interaction scores for a given pair versus the sum of the absolute differences in SMF. We observed a significant but weak correlation between SMF differences and changes in genetic interactions measured by our conditional z-score both in sorbitol (r=0.137, p-value<2.2×10^{-16}) and in oxidative stress (r=0.145, p-value<2.2×10^{-16}). In addition, we also counted the number of condition dependent conditional genetic interactions (abs(z-score)>2) for each array gene and observed a weak but significant correlation with the absolute change in SMF for the same genes both in sorbitol (r=0.11, p-value=0.0003) and oxidative stress (r=0.13 , p-value=4.52×10^{-5}). These observations tend to be condition specific since the correlations of SMF changes in one condition with the number of conditional genetic interactions in a second condition become even weaker. This is observed in SMF in oxidative stress versus the number of conditional genetic interactions in sorbitol (r=0.065,p-value=0.03831) and SMF in sorbitol versus the number of conditional genetic interactions in oxidative stress (r=0.04, p-value = 0.1648). These observations suggest that the changes in SMF are significantly associated with the changes in genetic interactions in the same stress but these account for a small fraction of the total variation observed. We also would not expect that the SMF differences would drive most of the changes in S-score across conditions since the S-scores are a measure of the deviation between the observed and the expected double-mutant growth in that condition. If there were only SMF differences and no differences in epistasis then the genetic interaction scores should not change. Our interpretation is that genes that show changes in SMF are more likely to have changes in functional interactions and that in turn results in higher changes in genetic interactions.

The reviewer correctly points out that the S-score is also a metric of confidence and suggests that differences in S-score between conditions could be due to systematic differences in confidence and not epistasis. A difference in confidence without a change in epistasis would be due to a change in variance in the replicates used to calculate the S-score. Such chances in S-scores are much less likely to be reproducible in replicates. In order to address this question and to further validate the conditional genetic interaction scores, we have re-generated a set of 31 double-mutants, including 21 pairs with *HOG1* and 10 with *SLT2*. We compared the viability of the single mutants and double mutants in stress and no stress using spot assays (now shown in supplementary figure 3). A total of 93 conditional genetic interactions were re-tested in this way. For all but 2 of the 31 pairs, the double-mutant did not appear to have a genetic interaction in absence of stress which is consistent with the no-stress S-score value for 90% of these pairs. We scored the change in genetic interaction in a qualitative schema with 5 groups from strongly conditional negative to strongly conditional positive (--/-/n/+/++). Two gene pairs showed negative-interactions in the absence of stress for the spot assays (*hog1-pho80* and *hog1-vps9*). For these we could not easily score the change in genetic interaction in sorbitol or zymolyase in the spot assays. For all other gene-pairs we then compared the conditional-genetic interaction score with the phenotypes observed in the conditional viability assays. The median conditional z-scores were found to be correlated with the qualitative ranking of the condition viability spot assays with median values of -3.64 for strong negative, -0.72 for negative, -0.014 for neutral, 0.94 for positive and 2.27 for strongly positive (shown in supplementary figure 4). Using the cut-off defined in the manuscript (abs(z-score)>2), 69,6% (62/89) of the tested conditional genetic interactions showed a similar phenotype (positive, neutral or negative) as the viability assays. It is worth pointing out that visualization inspection of spot tests is obviously far less quantitative than S-scores derived from the genetic interaction screens, nonetheless, it is satisfying to see a set of the interactions confirmed using an orthogonal approach.
All of spot assays are now provided in supplementary figure 3 and we now describe these results in the main text. This analysis suggest that most of the S-score differences across conditions are due to changes in epistasis as defined by difference in observed versus expected growth of the double mutant.

2) On page 5, the authors use the differential GI maps to rank genes according to their involvement in the different stress responses. While this approach recapitulates the known functional associations between signaling genes and specific stress responses, one might wonder how much of

this information would have been provided by single mutant fitness data alone? Clearly, SMF should also change across conditions, and it remains unclear how much more information is provided by measuring double mutant fitness as well. It would be important to convince the readers that measuring GI maps across conditions captures much better condition-dependent functions than simply performing chemogenomic screens (which requires way less effort). In addition, the benchmark of the functional ranking of query genes is anecdotal and no statistics is provided.

In order to address the concern regarding the functional ranking of the query genes, we tested if there is a correlation between the SMF in a given condition and the number of differential interactions the gene has in the same condition. For each of the query genes, we counted the number of differential interactions in sorbitol, H_2O_2 and caffeine and compared this value with the single mutant fitness defect in appropriate conditions. We observed a significant correlation between the SMF defect and number of interactions in sorbitol (r=0.72, N=28, p-value=$7x10^{-6}$) and oxidative stress (r=0.52, N=28, p-value=0.0012) but not in caffeine (r=-0.04, N=27). However, there is a correlation between the SMF defect in rapamycin and number of interactions in caffeine (r=0.71, N=24, p-value=$5x10^{-5}$). These observations lend credence to the fact that the degree of differential interactions associates signalling genes with their respective pathways. However, these correlations are driven by a small number of genes. For example, excluding *HOG1* and *PBS2* in sorbitol, *SKN7* in oxidative stress and *TOR1* and *SLT2* in caffeine (versus rapamycin SMF) abolishes the correlations (r=-0.32, r=-0.26 and r=0.15 respectively). For the HOG pathway, we compared the ranking of known pathway members according to the number of differential interactions and SMF defect scores in osmotic stress. Some pathway members (*MSB2*, *NBP2* and *SHO1*) are among the top genes with most differential interactions but don't have a strong SMF defect. Similarly, *HOT1* and *SSK22* show a significant SMF defect but have fewer differential genetic interactions. Both of these approaches appear to provide complementary condition specific functional information regarding single genes. Some of the examples that we have verified in the follow up experiments would not have been predicted based on SMF information alone. For example, COMPASS gene deletion mutants do not show a strong SMF defect in the presence of zymolyase but show clear differential genetic interactions and functional phenotypes (Figure 3d and 3e).

Importantly, the differential interactions provide gene-gene associations that the conditional SMF do not. This is analogous to the difference in information obtained for gene deletion growth phenotypes versus genetic interactions in optimal growth conditions. Although slow growing gene deletion mutants typically have a larger number of negative genetic interactions (Costanzo et al. 2010) knowledge of the actual pairs of genes showing the interactions is much more informative than the gene deletion growth phenotype. As we show in the last section of the manuscript the kinase-target interactions of the CWI and HOG pathways are significantly enriched in the differential genetic interactions in sorbitol and zymolyase (Figure 5c). This illustrates how these networks capture condition dependent functional associations between pairs of genes, something that cannot be obtained from SMF information alone. As we discussed in one of the points raised by reviewer 2, we currently lack systematic studies of condition dependent physical interactions. We think that such information would have allowed us to further highlight the benefit of having conditional genetic interaction data versus SMF defects. However, these data, along with other such studies, should spur further studies of condition dependent physical interactions.

Minor points:
1) Some relevant citations have been ignored, most notably the Costanzo et al. Science 2010 paper on the use of GI profiles as a measure of functional relatedness. Another example is Harrison et al. PNAS 2007 paper on the condition-dependency of GI in metabolism.

We have tried to improve the introduction section and have added the recommended citations.

2) The observation that genes with differential interactions did not overlap with genes that are differently expressed could be linked to the finding that up/downregulation of a gene is usually not correlated with its change in importance (see Giaever 2002 Nature, Price et al. MSB 2013)

In line also with the comments from another reviewer we have expanded the discussion regarding the low overlap between conditional genetics and conditional gene-expression.

3) Fig 3A: color coding is confusing. Zymolase seems to be yellow / orange, not red as indicated in the legend. See also caffeine.

We agree with the reviewer that the colour scheme in this figure can be confusing. We have removed the colour gradient for figure 3A.

4) page 10, methods of identifying gene-complex associations. The method is not very clearly presented, I had to read the caption of Fig 3 to fully grasp it. Also, it's confusing why GO-TermFinder was used here - I guess it was for calculating the stats, not to perform GO analyses. The statistical test employed should be explicitly stated.

We thank the reviewer for point this out. We have improved the description of the approach used in the methods section. We also describe the statistical test used to determine the significance of the enrichment tests as implemented in GO-TermFinder.

2nd Editorial Decision 24 February 2015

Thank you again for submitting your work to Molecular Systems Biology. We have now heard back from the referees who agreed to evaluate your manuscript. As you will see below, the reviewers are satisfied with the modifications made and only list a few remaining concerns which we would ask you to address in a revision of this work. Introducing sub-sections with descriptive titles in the results section (as reviewer #2 suggests) would indeed make the main findings better accessible.

Reviewer #1:

The authors have addressed most of the referees' concerns. However the continued use of the 'S-score' is still not very satisfying. I agree with the other referee that it would be better and more transparent to present separately the magnitude of the change in interaction/epistasis (effect size) and the confidence in this change (p-value). The authors have provided indirect evidence that the changes in S-scores are not due to changes in single gene effects, but it would also be good to directly measure this using standard measures of epistasis.

Reviewer #2:

The authors have addressed my earlier concerns. One minor comment: the manuscript would be more readable if the first section of Results section were broken into subsections with helpful headings-- they have added a number of experiments, which has made this section quite long.

Reviewer #3:

The authors adequately addressed all my criticisms / questions.

2nd Revision - authors' response 16 March 2015

We thank you and the reviewers for the additional comments. We are pleased that the revised version addresses the concerns raised by the reviewers. As suggested by one of the reviewers we

have split the first part of the Results section into subsections with corresponding summary headings. See below the response to the additional remarks from the reviewers.

Response to the reviewers.

Reviewer #1:

The authors have addressed most of the referees' concerns. However the continued use of the 'S-score' is still not very satisfying. I agree with the other referee that it would be better and more transparent to present separately the magnitude of the change in interaction/epistasis (effect size) and the confidence in this change (p-value). The authors have provided indirect evidence that the changes in S-scores are not due to changes in single gene effects, but it would also be good to directly measure this using standard measures of epistasis.

The S-score is a metric that reflects both the strength of interaction and the confidence in the observed genetic interactions. Although we understand the interest in having separate measures the effect sizes alone would be very prone to measurement variation. This measure of genetic-interaction has been extensively validated and we refer the reviewer and the readers to the detailed description provided in Collins et al. Genome Biology 2006.

Reviewer #2:

The authors have addressed my earlier concerns. One minor comment: the manuscript would be more readable if the first section of Results section were broken into subsections with helpful headings-- they have added a number of experiments, which has made this section quite long.

We agree with the reviewer and have split the first part of the Results section into subsections with corresponding summary headings.

© European Molecular Biology Organization
